# MiR-103 Controls Milk Fat Accumulation in Goat (*Capra hircus*) Mammary Gland during Lactation

**DOI:** 10.1371/journal.pone.0079258

**Published:** 2013-11-11

**Authors:** Xianzi Lin, Jun Luo, Liping Zhang, Wei Wang, Deming Gou

**Affiliations:** 1 Shaanxi Key Laboratory of Molecular Biology for Agriculture, College of Animal Science and Technology, Northwest A&F University, Yangling, Shaanxi, China; 2 Department of Biological Sciences, University of Alabama, Tuscaloosa, Alabama, United States of America; 3 College of Life Sciences, Shenzhen University, Shenzhen, Guangzhou, China; University of Cordoba, Spain

## Abstract

Milk is the primary source of nutrition for young mammals including humans. The nutritional value of milk is mainly attributable to fats and proteins fractions. In comparison to cow milk, goat milk contains greater amounts of total fat, including much higher levels of the beneficial unsaturated fatty acids. MicroRNAs (miRNAs), a well-defined group of small RNAs containing about 22 nucleotides (nt), participate in various metabolic processes across species. However, little is known regarding the role of miRNAs in regulating goat milk composition. In the present study, we performed high-throughput sequencing to identify mammary gland-enriched miRNAs in lactating goats. We identified 30 highly expressed miRNAs in the mammary gland, including miR-103. Further studies revealed that miR-103 expression correlates with the lactation. Further functional analysis showed that over-expression of miR-103 in mammary gland epithelial cells increases transcription of genes associated with milk fat synthesis, resulting in an up-regulation of fat droplet formation, triglyceride accumulation, and the proportion of unsaturated fatty acids. This study provides new insight into the functions of miR-103, as well as the molecular mechanisms that regulate milk fat synthesis.

## Introduction

Milk, one of the most complete foods in nature, is the primary source of nutrition for young mammals (including human beings). The major nutritional value of milk is attributable to fats and proteins, the first one being its most variable component [1]. In milk, almost 99% of fat exists in the form of fat globules, which are essentially a complex mixture of lipid droplets enclosed within a plasma membrane and secreted by mammary gland epithelial cells [2], [3]. Triglycerides synthesized from numerous fatty acids in mammary gland epithelial cells are the major type (>95%) of lipids present in milk fat globules [4], [5].

In comparison to cow milk, goat (*Capra hircus*) milk contains much higher levels of fatty acids, as well as higher levels of fats, proteins, carbohydrates, calcium, and vitamins [6]. Moreover, the composition of the fatty acids found in goat milk varies considerably to cow milk. Specifically, goat milk contains higher contents of long-chain fatty acids than cow milk [6]. Additionally, goat milk contains a much higher proportion of the short and medium chain fatty acids in comparison to cow milk [7], and it has been reported that these short and medium chain fatty acids are beneficial in the treatment of human dyspepsia and gastrointestinal dysfunction [8]. Furthermore, goat milk also contains greater amounts of unsaturated fatty acids than cow milk [5], [6], some of which (e.g., c9-C18:1, c6,9,12-C18:3 and c9,t11-C18:2) are thought to be functional for human health [9], [10]. Consequently, goat milk is suspected to have therapeutic value for many disorders and human diseases [9], [10]. However, we still know little about the genetic and molecular mechanisms that underlie the synthesis and regulation of goat milk. Improving milk quality through the alteration of milk composition to maximally benefit human health is one of the major goals of milk production. Therefore, understanding these mechanisms may lead to novel strategies resulting in further improving the nutritional values of goat milk and genetically modify the milk composition.

MicroRNAs (miRNAs) are endogenous single stranded non-coding RNAs of about 22 nucleotides (nt) in length. MiRNAs have been shown to have significant regulatory roles by targeting mRNAs for translational repression or cleavage [11], [12], [13]. The pairing between miRNA and target mRNA is mainly dependent on the 6–8 nt seeding sequences of the miRNA, and as a result each miRNA is predicted to target numerous target genes [13], [14]. MiRNAs have been demonstrated as regulators in tissue development [15], [16], cell differentiation [17], [18], lipid metabolism [19], [20], immune response [21], [22], and oxidative stress response [23]. Mammary glands synthesize lipids and secret milk fat upon successive cycles of adult development and lactation. From pregnancy to lactation, the mammary gland exhibits different physiologicaland biological statuses [24], [25]. Recent studies have provided important clues suggesting that miRNAs are involved in the development and lactation of the mammary gland. In mice, miR-101a controls mammary gland development by regulating cyclooxygenase-2 expression [15]. MiR-205 over-expression leads to an expansion of the progenitor-cell population and increased cellular proliferation [26], while miR-27 reduces lipid accumulation by targeting peroxisome proliferator-activated receptor γ (PPARγ) in human adipocyte cells [27], and miR-33 represses sterol transporters in human liver cells [28]. These results suggest that miRNAs in goats may be crucial for gene regulation in mammary gland development and lactation. Recent studies have identified miRNA profiles in the mammary glands of the Chinese Laoshan dairy goat (a native dairy goats breed) during early-lactation by high-throughput sequencing [29]. However, these results could not fully reflect the miRNA profiles in lactation. Lactation cycle can be divided into three periods: early-, mid- and late-lactation. During mid-lactation, milk yield is higher than in the other periods. Furthermore, studies have shown that miRNA profiles in early-lactation differ in comparison to mid-lactation of mouse mammary glands [30]. Therefore, it is necessary to screen and identify the miRNAs involved in the mid-lactation of dairy goats.

Solexa sequencing, a high-throughput sequencing approach is an optimal method to identify unknown molecule sequences, including miRNAs, and accordingly, many universal and novel miRNAs have been identified using this method in various species, such as cabbage [31], urchin [32], pig [33], sheep [34], and human [35]. Furthermore, Solexa sequencing can reflect the miRNA relative abundance by number of read counts of miRNAs [34], [35].

In this study, we used Solexa sequencing to profile all miRNAs present in goat mammary glands. Through miRNA abundance analysis, we obtained mammary gland-enriched miRNAs. From abundantly expressed miRNAs, we chose miR-103, which has been reported to be involved in lipid metabolism in adipose tissue, to further investigate the correlation between this miRNA and lactation. To this end, we generated a recombinant adenovirus expressing miR-103 (Ad-miR-103), and investigated the effect of elevated expression of miR-103 on milk fat synthesis using goat mammary gland epithelial cells (GMEC). Our results indicate that miR-103 has a significant role in milk fat accumulation in goats. This is the first study which demonstrates that miRNAs participate in milk fat synthesis, and provides further insights into miRNAs functions in lactating goats.

## Results

### Identification of miRNAs by Solexa sequencing in goat mammary glands at mid-lactation

To identify miRNAs involved in lactating goats, mammary gland tissue was harvested from 10 individuals during mid-lactation (120 days after parturition) and subjected to RNA extraction. These RNA samples were pooled and used for Solexa sequencing. A small RNA library was constructed and sequenced using Illumina Genome Analyzer Pipeline (Illumina, San Diego, USA). We yielded 22,084,321 reads count of small RNAs. The 21–23 nt RNAs are the majority of small RNAs (Figure S1). The products of Dicer-processing are mainly 21–23 nt miRNAs [12], suggesting that the majority of small RNAs in goat mammary gland may be miRNAs. Subsequently, all the small RNAs were then mapped to various established small RNA libraries. After discarding other small RNAs (14.3%) (i.e., tRNA, rRNA) (Table S1), 12,367,141 reads accounted for 47.7% of the total small RNAs obtained. The high percentage composition of miRNAs indicates that miRNAs may represent the majority of small RNAs involved in gene regulation in goat mammary glands during mid-lactation. Then, we aligned the obtained sequences with current annotated miRNAs in the miRBase. (Release 17.0) (no goat miRNAs in the database). MiRNAs corresponding to known mammalian miRNA were considered to be conserved miRNAs in this study.

**Figure 1:**
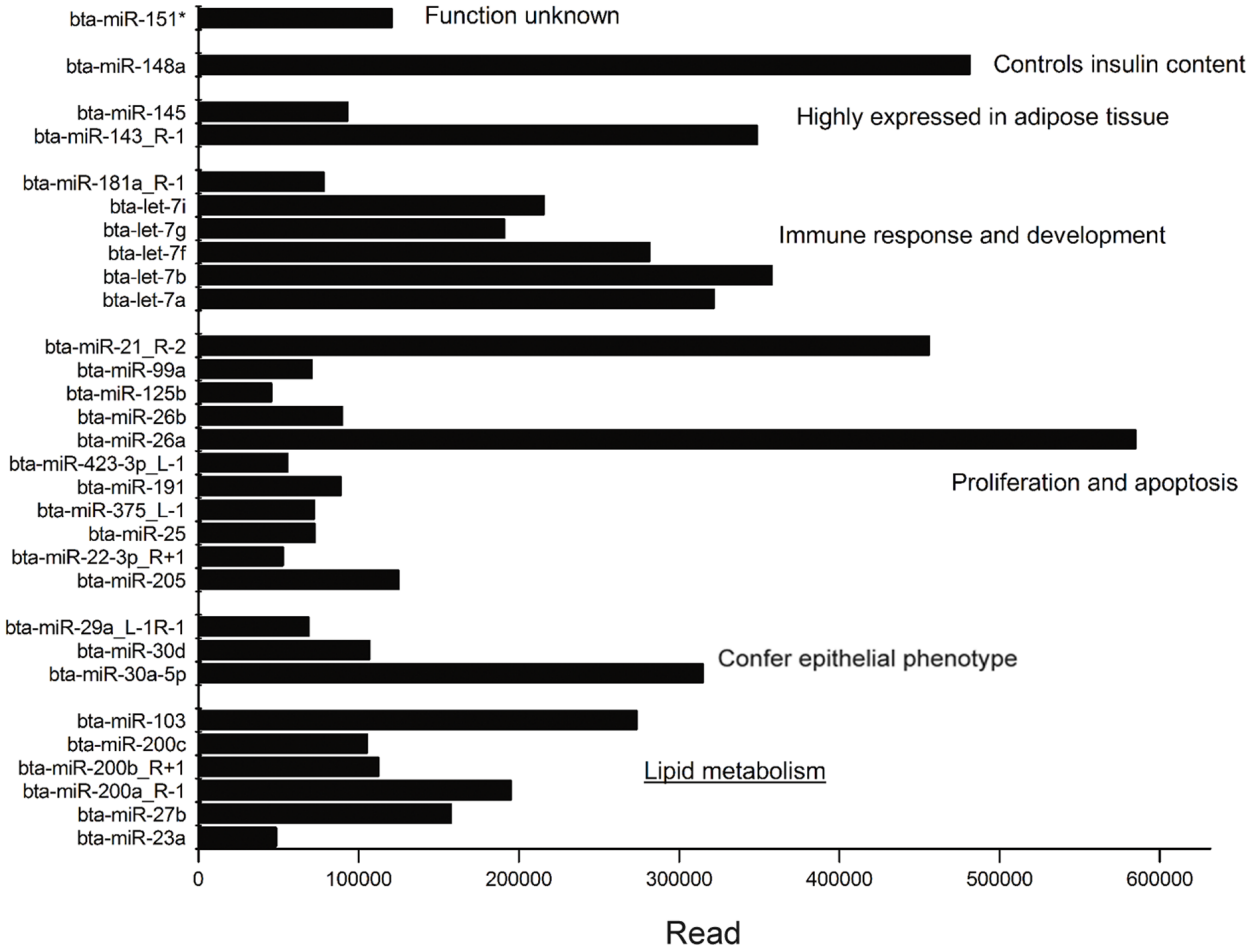
The top 30 miRNAs with maximum number of reads count in goat mammary gland during mid-lactation. RNA samples were pooled from 10 individuals during mid-lactation (120 days after parturition). The top 30 miRNAs in mammary gland were identified by Solexa sequencing and clustered based on their reported function (Table S2). Values on X-axis indicate the number of reads count of miRNAs. All goat miRNAs are named after bovine (*Bos taurus*). “R+1” and“L-1”: compared to bovine's sequence, goat miRNA sequence has addition or reduction of one base on the 3′ or 5′end.

823 conserved miRNAs were identified and subjected to the estimation of their relative abundance. Interestingly, we found that the top 30 most abundantly expressed miRNAs constitute 21.18% of the total miRNAs and they are all conserved miRNAs. We categorized these 30 miRNAs into seven clusters based on their previously reported functions (Figure 1 and Table S2), which included proliferation and apoptosis, immune response and development, lipid metabolism, and epithelial phenotype conferring. Prior to lactation, mammary glands proliferate at a high rate to generate a vast number of epithelial cells [25]. During lactation, mammary gland epithelial cells are responsible for milk fat synthesis and fat globule secretion, which is regulated by the action of several hormones (i.e., insulin) [36]. In the mammary glands of lactating goats, we found that miRNAs associated with cell proliferation (miR-26a, miR-21), conferring epithelial phenotype (miR-29a, miR-30a/d), immune response and development (miR-181, let-7a/b/f/g/i) were abundantly expressed, as well as miRNAs involved in lipid metabolism (miR-103, miR-23a, miR-27b, miR-200a/b/c). Specifically, miR-26a (cell proliferation) was the most abundantly expressed, followed by miR-148 (may control insulin content) and miR-21 (cell proliferation). These data suggest that miRNAs play a critical role in mammary gland regulation and cell proliferation, as well as immune responses, and lipid metabolism. Specific knockdown of miR-148 in pancreatic β-cells or in isolated primary islets down-regulates insulin mRNA levels [37]. Mammary gland does not synthesize insulin, therefore miR-148 is not included in the “lipid metabolism” cluster. Moreover, miR-143 and miR-145, are highly expressed in adipose tissue, and are also abundantly expressed in the goat mammary glands as well, suggesting the same regulatory function of some miRNAs in mammary glands and adipose tissue.

The regulation of lipid metabolism within the mammary glands is a complex process requiring precise regulation of fatty acid, triglyceride, and cholesterols synthesis, all of which are necessary for lactation [2], [3]. However, genetic and molecular mechanisms that control this complex process in goats are barely known, especially the roles that miRNAs play. From the top 30 miRNAs, we found six miRNAs (e.g., miR-23a, miR-27b, miR-103, miR-200a/b/c) to be related to lipid metabolism in human adipocyte cells: miR-23 enhances glutamine metabolism [38]; miR-27 decreases fat accumulation [27]; miR-103 regulates triglyceride content during cell differentiation [39]; and miR-200 affects insulin signaling [40]. Of the six miRNAs evaluated, miR-103 is the most abundantly expressed miRNA (272,319) (Table S2). In addition, some predicted targets of miR-103 (i.e., Long-chain acyl-CoA synthetase 1[ACSL1]) are involved in lactation [41]. Thus, miR-103 was chosen for further functional studies.

### MiR-103 is differentially expressed in mid-lactation and dry period

**Figure 2:**
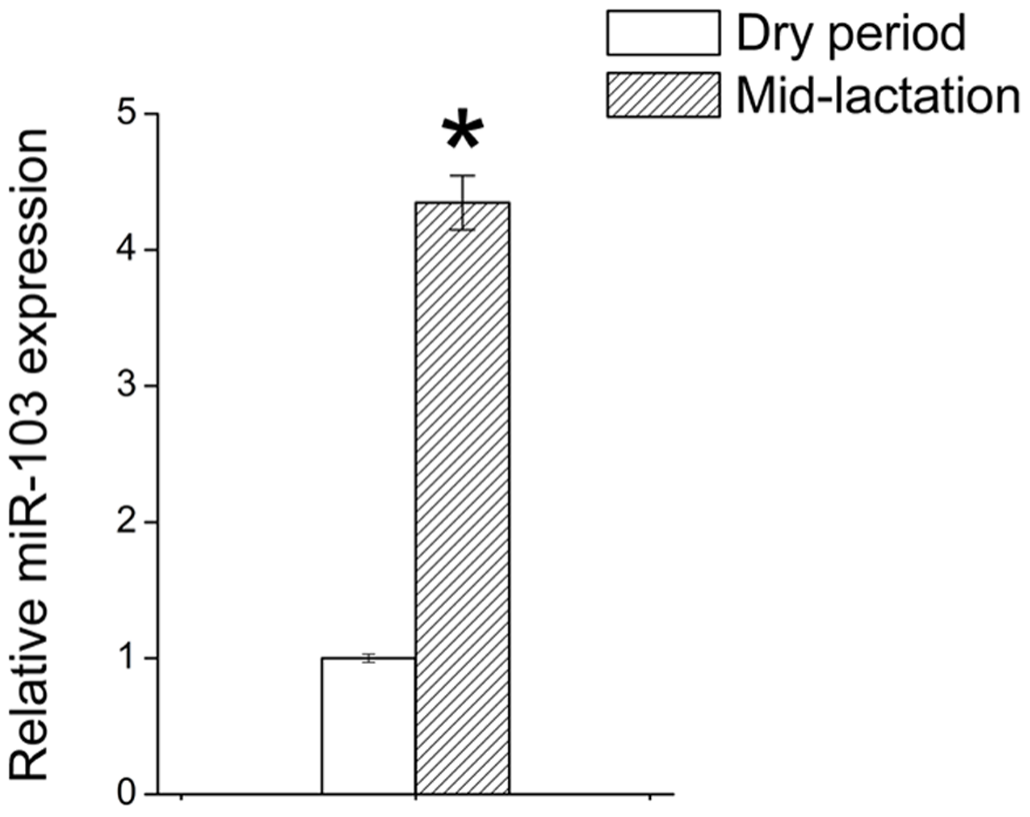
MiR-103 expression correlates with lactation stages. MiR-103 is differentially regulated at mid-lactation and dry period. qRT-PCR measurement of miR-103 level at mid-lactation expressed as fold change compared to that at dry period, normalized to 1. Columns, average of 12 experiments; bars, SEM. *, *p*<0.05.

The mammary gland undergoes extensive changes in tissue structure and milk production between the dry period and lactation. The enrichment of miR-103 at the mid-lactation stage may be a reflection of its physiological role in the regulation of lactation. Mammary gland tissue, randomly sampled from three goats during mid-lactation (120 d after parturition) and the dry period (60 d before parturition), was used for RNA extraction, and pooled RNA samples for further analysis. We compared miR-103 expression in mammary gland at two different physiological stages by quantitative Real-time PCR (qRT-PCR) and found the expression level of miR-103 was higher (4.3-fold, *p*<0.05) during the mid-lactation period than that in the dry period (Figure 2), suggesting that miR-103 may be involved in regulating lactation or development of the adult mammary gland.

### MiR-103-1 transcriptionally regulates its co-regulated host gene PANK3

Previous studies have shown that miR-103 is generally expressed in different tissues and cells [42], [43], [44], while differentially expressed during adipogenesis [45] and development [46]. Ectopic expression of miR-103 in preadipocyte 3T3-L1 cells up-regulated adipogenesis markers and increased triglyceride accumulation at an early stage of adipogenesis [47], while miR-103 silencing in OB/OB mice resulted in reduced levels of fat-pad weights [48]. The MiR-103 family has three members, miR-103-1, miR-103-2 and miR-107, which reside in the sense oriented intron 5 of three members of the pantothenate kinase (PANK) gene family members across species: *PANK3*, *PANK2*, and *PANK1*, respectively. PANK enzymes catalyze the rate-limiting step in Co-enzyme A (CoA) synthesis, and are thus considered to be important metabolic regulators [49], [50], as CoA is a necessary cofactor for enzymatic reactions including important steps in the synthesis of fatty acids, amino acids, cholesterol, pyruvate/lactate, glucose, and Krebs cycle intermediates [49].

**Figure 3:**
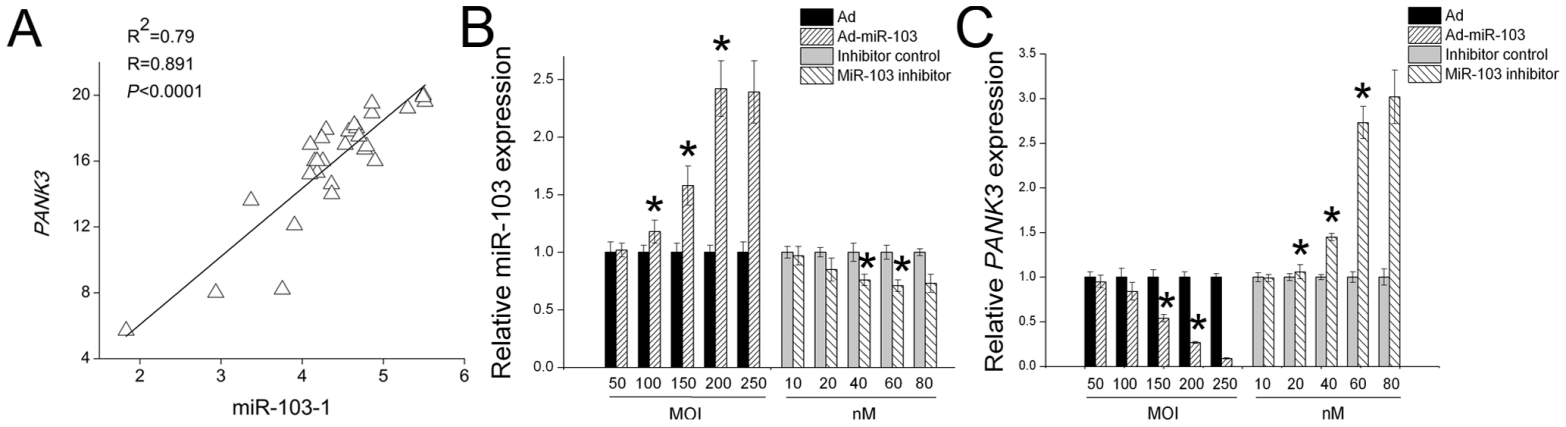
MiR-103-1 transcriptionally regulates its co-regulated host gene *PANK3*. A: A positive correlation between miR-103-1 and *PANK3* expression in mammary gland of 30 lactating goats. This correlation suggests that miR-103-1 and its host gene *PANK3* are co-regulated in goat. Intensity scatter plot shows comparison of expression of miR-103 and *PANK3*; B: The optimal MOI (200) of Ad-miR-103 for infection and dose of miR-103-inhibitor for transfection (60 nM). Ad-control without any miRNAs sequences or inhibitor-control was used as control. GMEC was used as a model. The data (miR-103 levels) were expressed as fold change as compared to controls, normalized to 1. Columns, average of 12 experiments; bars, SEM. *, *p*<0.05; C: MiR-103-1 transcriptionally regulates its host gene *PANK3*. Over-expression of miR-103 down-regulates the expression of PANK3 in GMEC, whereas suppression of miR-103 leads to up-regulation of *PANK3*. Over-expression or suppression of miR-103 was conducted by using Ad-miR-103 or miR-103 inhibitor as controls, respectively. MOI of Ad-miR-103 and concentration of inhibitor is shown under X coordinate axis. The data (*PANK3* levels) were expressed as fold change as compared to controls, normalized to 1. Columns, average of 9 experiments; bars, SEM. *, *p*<0.05.

Previous studies have shown that some sense oriented intronic miRNAs are co-regulated with their host genes; these miRNAs can positively assist the function of their co-regulated host genes [51], [52]. For example, miR-33, which is a sense oriented intronic miRNA in the sterol regulatory element-binding protein (SREBP), is co-regulated with *SREBP* and is capable of targeting ATP-binding cassette sub-family G (ABCG1) [52] which is downstream of SREBP [53]. MiR-103-1 resides in the sense oriented intron 5 of *PANK3* which is an important enzyme for fatty acid synthesis [49], [50]. To investigate whether miR-103-1's function is connected with *PANK3*'s, we must know whether miR-103-1 expression is co-regulated with *PANK3*'s. We compared their expression in the mammary glands of 30 goats by Pearson correlation and found a strong correlation between miR-103-1 and *PANK3* (R = 0.891, *p*<0.001) (Figure 3A). This result is consistent with previous studies done in the mouse 3T3-L1 cell line [47], suggesting that co-regulation of miRNA-103 and its host gene is highly conserved in mammals.

As the transfection efficiency with recombinant plasmids in GMEC was very low (< 5%), we used a recombinant adenovirus construction, which has been shown to be a highly effective and safe method to over-express genes in recalcitrant cell lines [54], [55]. Interestingly, we found that over-expression of miR-103 using Ad-miR-103 (adenovirus inserted with miR-103) down-regulates the expression of *PANK3*, whereas suppression of miR-103 leads to up-regulation of *PANK3* (Figure 3B, C) by using miR-103-antisense-inhibitor, a small, chemically modified single-stranded RNA molecule designed to specifically bind to and inhibit endogenous miRNA molecules. Additionally, no miR-103 binding sites were found in the 3′, 5′or the coding region of *PANK3*, indicating that this regulation is indirect. A possible explanation is that miR-103 may target other genes that can affect the transcriptional activity of *PANK3*. Taken together, these data imply that miR-103-1 transcriptionally regulates its co-regulated host gene *PANK3* in goat.

### Over-expression of miR-103 promotes milk fat droplet accumulation in GMEC

**Figure 4:**
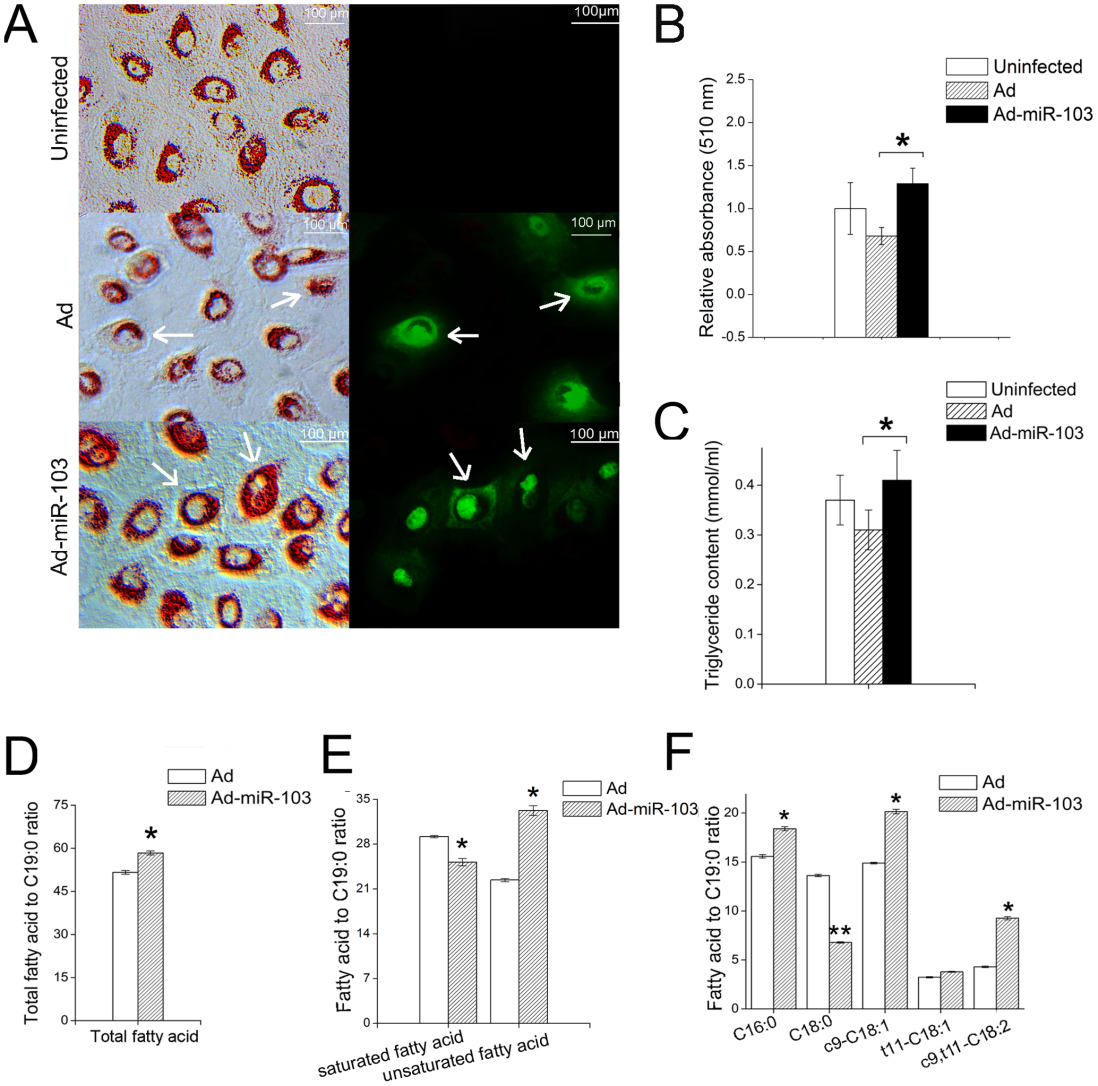
MiR-103 promotes milk fat accumulation in GMEC. A: Over-expression of miR-103 promotes fat accumulation. Cells in the left column were stained by Oil Red. The fluorescence of the cells was in the right column. Uninfected (first row) and Ad-infected cells (second row) were both used as controls. Arrows indicate Ad- and Ad-miR-103-infected cells; B: Over-expression of miR-103 increases fat droplet content. Fat droplets were extracted using isopropanol in un-, Ad-control without any miRNA sequences and Ad-miR-103-infected cells at 72 h post infection. The data (absorbance, 510 nm) were expressed as fold change as compared to uninfected cells, normalized to 1. Columns, average of 3 experiments; bars, SEM. *, *p*<0.05; C: Elevated miR-103 expression up-regulates triglyceride content. Triglyceride content was determined by using a Serum Triglyceride Determination Kit in un-, Ad-(control) and Ad-miR-103-infected cells. Columns, average of 12 experiments; bars, SEM. *, *p*<0.05; D: Over-expression of miR-103 increases total fatty acids content in GMEC. Fatty acid content expressed as fold change compared to internal control C19:0. Columns, average of 9 experiments; bars, SEM. *, *p*<0.05; E: Over-expression of miR-103 reduces total saturated fatty acids and promotes total unsaturated fatty acids. Columns, average of 9 experiments; bars, SEM. *, *p*<0.05; F: Over-expression of miR-103 alters the composition of major types of fatty acids in GMEC. Columns, average of 9 experiments; bars, SEM. *, *p*<0.05, **, *p*<0.01.

*In vivo*, the fat droplet in GMEC is secreted outside the cell to form the milk fat globule [3]. We compared the fat droplet formation between cells over-expressing miR-103 and controls that included cells infected by adenovirus (Ad) without any miRNA sequences and uninfected cells by using oil red O. With an optimal dose (multiplicity of infection: 200), we found that over-expression of miR-103 causes significantly increased fat droplet accumulation compared to Ad-control and uninfected cells (Figure 4A, B). However, no significant differences were observed between the fat droplet accumulation in cells treated with the miR-103-inhibitor and the negative control (data not show). One possible explanation is that the suppression of miR-103 could be compensated by other redundant signaling pathways and thereby, knockdown of miR-103 alone does not affect fat droplet formation.

### MiR-103 increases triglyceride accumulation in GMEC

Milk fat globules are composed almost exclusively of triglycerides [4], [5]. We used a triglyceride determination kit (Sigma-Aldrich) to determine the triglyceride content in GMEC at 72 h after infection with Ad-miR-103 virus. The uninfected and Ad without any miRNA sequences -infected cells were used as controls. In control groups, we found that triglyceride content of Ad-infected cells was slightly lower than that of uninfected cells (Figure 4C), suggesting that adenovirus may affect triglyceride accumulation. Triglyceride content of Ad-miR-103-infected cells was higher than that of uninfected and Ad-infected cells (Figure 4C). Specifically, triglyceride content of Ad-miR-103-infected cells was 33% higher (*p*<0.05) than that of Ad-infected cells. Our data suggests that miR-103 plays an important role in regulating triglyceride synthesis.

### Elevated miR-103 expression alters fatty acid composition in GMEC

Fatty acids are stored in the form of triglycerides in epithelial cells [3], [4], and in milk they are further clustered as saturated and unsaturated fatty acids, or cis- and trans-fatty acids [6], [9]. We harvested GMEC at 72h post-infection by Ad-(control) and Ad-miR-103, respectively. Cells were methyl-esterified, and the components and contents of fatty acids were analyzed by gas chromatography-mass spectrometry (GC-MS). Compared to the Ad-infected cells, the total fatty acid content in Ad-miR-103-infected cells was significantly greater (1.16-fold, *p*<0.05) (Figure 4D). A significant decrease was found in the total saturated fatty acids (0.86-fold, *p*<0.05) (Figure 4E), whereas, a marked increase was observed in the total unsaturated fatty acids (1.48-fold, *p*<0.05) (Figure 4E), resulting in an up-regulated ratio of unsaturated/saturated fatty acids. The analysis of fatty acid contents showed that Ad-miR-103-infected cells accumulated more c9-C18:1 (1.35-fold, *p*<0.05), t11-C18:1 (1.17-fold) and c9,t11-C18:2 (2.16-fold, *p*<0.05) (Figure 3F). Furthermore, C16:0 content of Ad-miR-103-infected cells was higher than that of the control (1.18-fold, *p*<0.05), whereas C18:0 was lower compared to the control (0.49-fold, *p*<0.01) (Figure 3G). These five types of fatty acids identified in GMEC are all common and important components of goat milk [6], [7], [8]. Specifically, c9-C18:1 and t11-C18:1 are unsaturated fatty acids which have been shown to decrease the risk of cardiovascular disease [7], [9]; c9,t11-C18:2 (linoleic acid) is known for its broad range of beneficial effects on human health [7], [9], [10]. Taken together, miR-103 plays a significant role in regulating fatty acid composition.

### MiR-103 up-regulates gene expression associated with the milk fat synthesis process in GMEC

Milk fat synthesis in mammary gland epithelial cell is controlled by complex gene networks which consisting of multiple metabolic processes including de novo fatty acid synthesis, triglyceride synthesis, fatty acid uptake, and fat droplet formation [56]. Fatty acids are de novo synthesized by fatty acid synthase (FASN) and acetyl-coenzyme A carboxylase alpha (ACACA) [57]. These fatty acids are then unsaturated by stearoyl-CoA desaturase (SCD) and processed into triglycerides by diacylglycerol acyltransferase1 (DGAT1) in the endoplasmic reticulum [56]. Fatty acids from outside the cell are hydrolyzed by lipoprotein lipase (LPL) and transported into cells by CD 36 molecule thrombospondin receptor (CD36) and solute carrier family 27 transporter sub-family A member6 (SLC27A6)[57], [58], [59]. These fatty acids are also incorporated into triglycerides within the endoplasmic reticulum. All triglycerides coalesce to form fat droplets by adipose differentiation related protein (ADRP) and PAT-related protein family member 47 (TIP47) [60], and subsequently secreted out by butyrophilin subfamily 1 member A1 (BTN1A1) [61]. To investigate how miR-103 affects milk fat synthesis, we assessed the expression of key genes involved in these processes at 72 h in GMEC that over-expressing miR-103 (Table 1). For de novo fatty acid synthesis, expression levels of *FASN* and *ACACA* in Ad-miR-103-infected cells were significantly higher than in control groups (Ad-infected cells) (1.15-fold, *p*<0.05; 1.57-fold, *p*<0.05). For fatty acid hydrolysis and uptake, expression of *LPL* and *SLC27A6* in Ad-miR-103-infected cells was significantly greater than that of Ad-infected cells (20.48-fold, *p*<0.01; 1.40-fold, *p*<0.05). Specifically, *SLC27A6* is responsible for long-chain and saturated fatty acid transport [59]. The up-regulation of *LPL* and *SLC27A6* are in accordance with the increased total fatty acid content (Figure 4D) and up-regulated C16:0 content (Figure 4F), suggesting that a higher level of fatty acid utilization is triggered by augmented miR-103 expression. For triglyceride synthesis, expression of *DGA1* (1.21-fold, *p*<0.05) and S*CD* (2.87-fold, *p*<0.05) was up-regulated with elevated miR-103 expression, supporting our previous findings that triglyceride content and unsaturated fatty acids content were both increased in epithelial cells (Figure 4C, E). Furthermore, for fat droplet formation, the up-regulated expression of *ADRP* (18.02-fold, *p*<0.05) was consistent with the increased fat droplets in Ad-miR-103-infected cells (Figure 4B). Taken as a whole, miR-103 has an extensive role in regulating milk fat synthesis in goats. The entire gene network that controlls the milk fat synthesis, was up-regulated, supporting our previous findings that miR-103 over-expression increased the amount of fat droplets, as well as the contents of triglycerides and fatty acids.

**Table 1:** Relative mRNA expression in miR-103 over-expression or suppression background.

Gene symbol	Gene description	Ad-miR-103 to Ad	MiR-103-inhibitor- to inhibitor-control
**De novo fatty acid synthesis**			
*FASN*	Fatty acids synthase	1.15*	0.67*
*ACACA*	Acetyl-coenzyme A carboxylase alpha	1.57*	0.88
*ACSS2*	Acyl-CoA synthetase short-chain family member 2	1.91	0.56
**Triglyceride synthesis**			
*SCD*	Stearoyl-CoA desaturase(delta-9-desaturase)	2.87*	0.83*
*GPAM*	Glycerol-3-phosphate acyltransterase	1.11	0.76
*AGPAT6*	1-acylaglycerol-3-phosphate O-acyltransferase 6	0.62	3.33
*LPIN1*	Lipin 1	0.50	3.11
*DGAT1*	Diacylglycerol acyltransferase 1	1.21*	0.83*
**Milk fat droplet formation and secretion**			
*ADFP*	Adipose differentiation related protein	18.02*	0.82*
*TIP47*	PAT-related proteins family, member 47	0.78	0.89*
*gBTN1A1*	Goat Butyrophilin, subfamily 1, member A1	0.97	0.94
**Fatty acid uptake**			
*LPL*	Lipoprotein lipase	20.48**	0.87
*CD36*	CD 36 molecule (thrombospondin receptor)	3.75	0.89
*SLC27A6*	Solutecarrier family27transporter,sub-family A,member6	1.40*	0.94
**Fatty acid transport**			
*ABCA1*	ATP-binding cassette,sub-family A, member 1	1.25*	1.02
*ABCG1*	ATP-binding cassette,sub-family G, member 1	1.15	1.13
*ABCG2*	ATP-binding cassette,sub-family G, member 2	1.21	1.19**
**Fatty acid intra-cellular transport**			
*FABP4*	Fatty acids-binding protein 4	0.81	1.18
**Fatty acid activation**			
*ACLY*	ATP-citrate lyase	0.50	1.64
*PANK3*	Pantothenate kinase family,member 3	0.32*	1.26**
*ACSS1*	Acyl-CoA synthetase short-chain family, member 1	1.91	1.09
*PDK4*	Pyruvate dehydrogenase kinase family, member 4	0.10*	2.48*
**Fatty acid receptor**			
*GPR41*	Orphan G protein-coupled receptor family, member 41	0.29	4.71

Genes were clustered based on main functions relative to milk fat synthesis. Ad(control)-infected cells were used as control for Ad-miR-103-infected cells. Inhibitor-control was used as control for miR-103-inhibitor-control. MRNA expression levels were determined at 72 h post infection or at 48 h post transfection. MRNA levels were measured by qRT-PCR, Data expressed as fold change compared to controls, normalized to 1. Columns, average of 12 experiments; *, *P*<0.05; **, *P*<0.01.

### Searching for the underlying causes of increased fat accumulation

**Figure 5:**
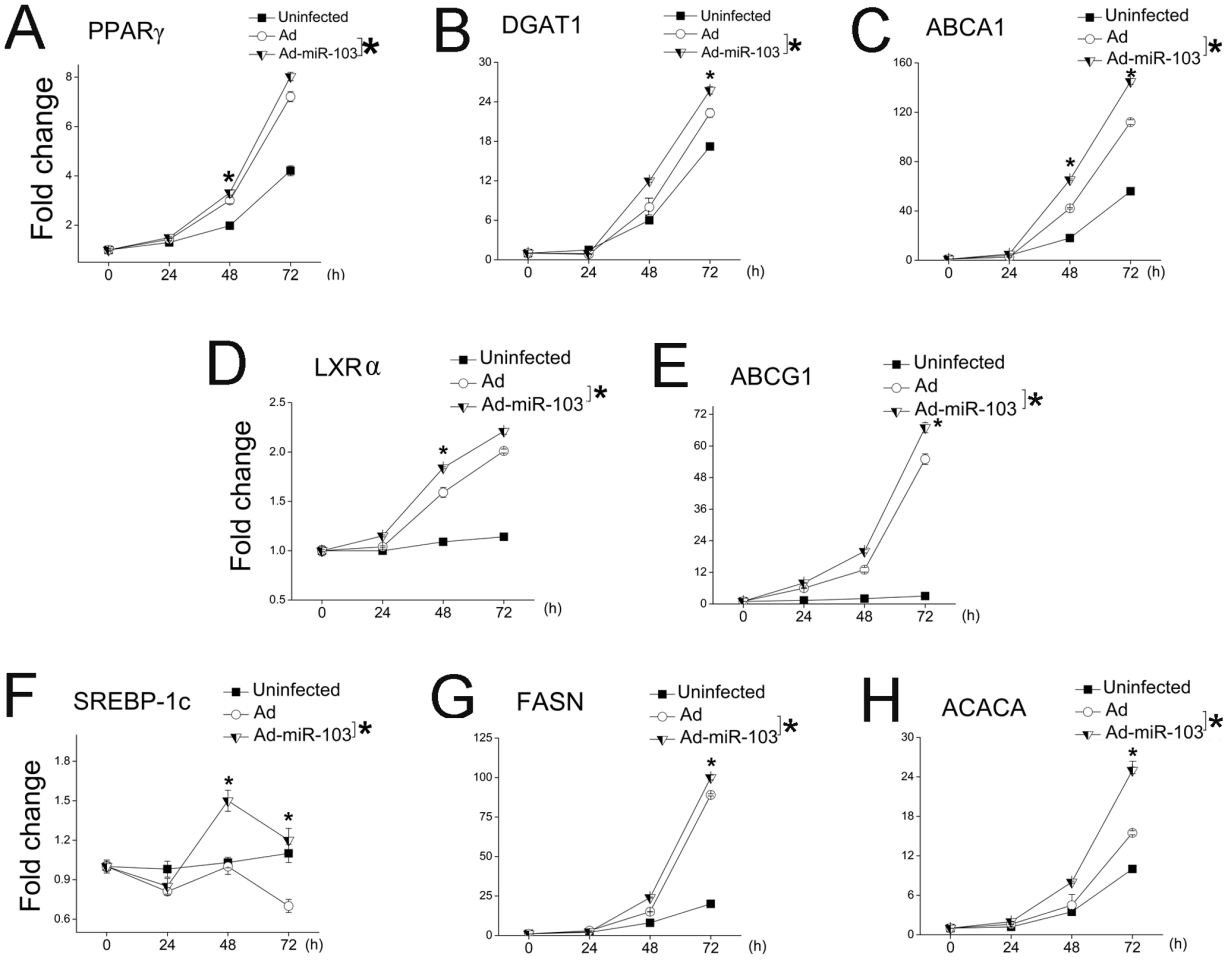
Over-expression of miR-103 transcriptionally hastens the expression of key transcription factors controlling the milk fat synthesis. Elevated miR-103 expression promotes the expression levels of *PPARγ* (A), *DGAT1*(B), *ABCA1*(C), *LXRα* (D), *ABCG1*(E), *SREBP-1c*(F) *FASN* (G), and *ACACA* (H). *PPARγ*, *SREBP-1c*, and *LXRα* are key important transcription factors that regulate nearly the whole milk fat synthesis process in mammary gland epithelial cells. *DGAT1*(B) and *ABCA1*(C) are downstream targets of PPARγ (A); *ABCG1*(E) is downstream target of LXRα (D); *FASN* (G) and *ACACA* (H) are downstream targets of SREBP-1c (F). Gene expression in Ad(control)-infected, Ad-miR-103-infected, and uninfected GMEC was assessed at 0, 24, 48, and 72 h. qRT-PCR measurement of gene expression expressed as fold change compared to their respective level at 0 h, normalized to 1. Columns, average of 12 experiments; bars, SEM. *, *p*<0.05. *PPARγ*, peroxisome proliferator-activated receptor γ; *SREBP-1c*, sterol regulatory element-binding protein-1c; *LXRα*,nuclear oxysterol receptorα;other gene symbols were listed in Table 1.

Previous studies have shown that PPARγ, SREBP-1c, and liver-X-receptor α (LXRα) are key transcription factors involved in the regulation of milk fat metabolism, by triggering the transcription of genes associated with triglyceride synthesis (PPARγ targeting *DGAT1*, and ATP-binding cassette sub-family A member 1 [*ABCA1*]), fat droplet formation (PPARγ targeting *ADRP*) [62], [63], cholesterol transport (LXRα targeting *ABCG1*) [64], and fatty acid synthesis (i.e., SREBP-1c targeting *FASN* and *ACACA*) [65]. These transcription factors and their downstreams are not predicted targets of miR-103. We assessed the mRNA expression of these three transcription factors at 0, 24, 48, and 72 h in GMEC with Ad-miR-103 (Figure 5) (the data of Figure 5 (72 h) and Table 1 were generated from a same experiment). Ad-infected cells were used as a control. Throughout the observation period, the rise of *PPARγ* expression (Figure 5A) paralleled the elevation of *DGAT1* expression (Figure 5B) and *ABCA1* expression (Figure 5C). The up-regulated expression of *LXRα* (*p*<0.05; Figure 5D) was consistent with the increased expression of *ABCG1* (*p*<0.05; Figure 5E). *SREBP-1c* (Figure 5F) showed a different expression profile with *FASN* (Figure 5G) and *ACACA* (Figure 5H); however, the expression of these three genes in Ad-miR-103-infected cells was always greater than that of Ad-infected cells. Based on the similar expression profiles of transcriptional factors and their downstream targets, we speculated that up-regulation of genes associated with the milk fat synthesis in miR-103 over-expression background may be due to the increased expression of *PPARγ*, *SREBP-1c*, and *LXRα* in GMEC.

**Figure 6:**
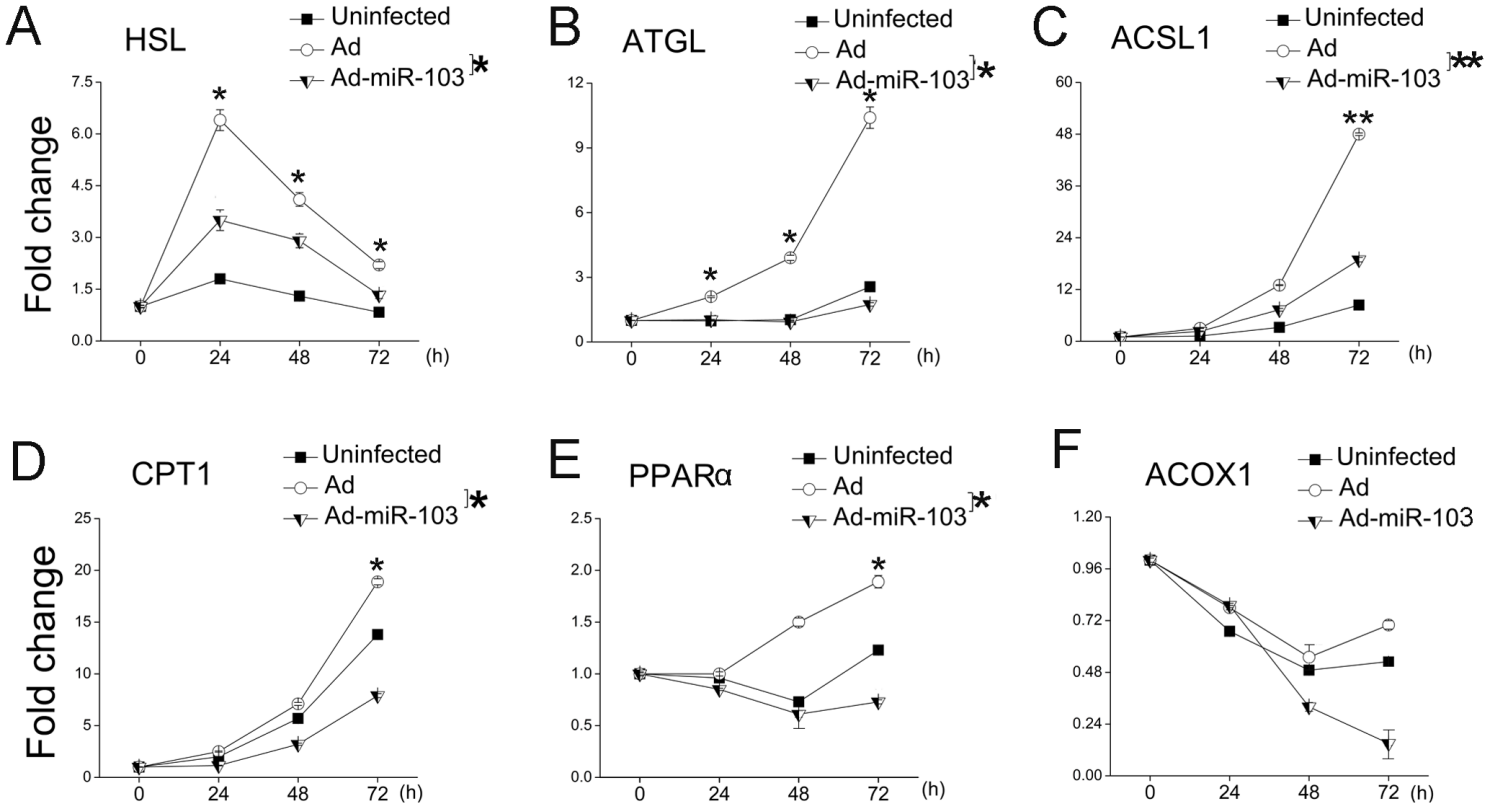
Elevated miR-103 expression decreases gene expression associated with lipolysis and β-oxidation. Over-expression of miR-103 down-regulates the mRNA levels of *HSL* (A), *ATGL* (B), *ACSL1*(C), *CPT1* (D), *PPARα* (E) and *ACOX1* (F). *HSL* (A) and *ATGL* (B) are key regulators of lipolysis. *ACSL1*(C), *CPT1* (D), *PPARα* (E) and *ACOX1* (F) are key regulators of β-oxidation. Gene expression in Ad(control)-infected, Ad-miR-103-infected, and uninfected cells was assessed at 0, 24, 48, and 72 h. qRT-PCR measurement of gene expression expressed as fold change compared to their respective level at 0 h, normalized to 1. Columns, average of 12 experiments; bars, SEM. *, *p*<0.05, **, *p*<0.01.

Suppression of lipolysis or β-oxidation can accelerate triglyceride accumulation in adipose tissue [66], [67]. For lipolysis, two enzymes (i.e., hormone-sensitive lipase [HSL] and adipose triglyceride lipase [ATGL]) catabolize triglycerides stored within lipid droplets to release fatty acids and glycerol [68]. Fatty acids produced from lipolysis are transported by long-chain acyl-CoA synthetase 1 (ACSL1, a predicted target of miR-103 [Table S3]), enter into mitochondria by carnitine palmitoyltransferase (CPT1), and undergoes β-oxidation which is regulated by peroxisome proliferator-activated receptor α (PPARα) and acyl-CoA oxidase 1 (ACOX1, a predicted target of miR-103 [Table S3]) [41]. In this study, *HSL* and *ATGL* expression was lower than that of the Ad control through the observation period (Figure 6A, B). The expression profiles of *ACSL1*, *CPT1*, *PPARα*, and *ACOX1* were quite different; however their expression in Ad-miR-103-infected cells was lower than the Ad control (Figure 6C-F). Taken together, the changes in gene expression have led us to conclude that either the augmentation of gene transcription or the decreased lipolysis and β-oxidation levels, or both, accelerate triglyceride accumulation in GMEC.

## Discussion

### MiR-103 regulates milk fat synthesis

**Figure 7:**
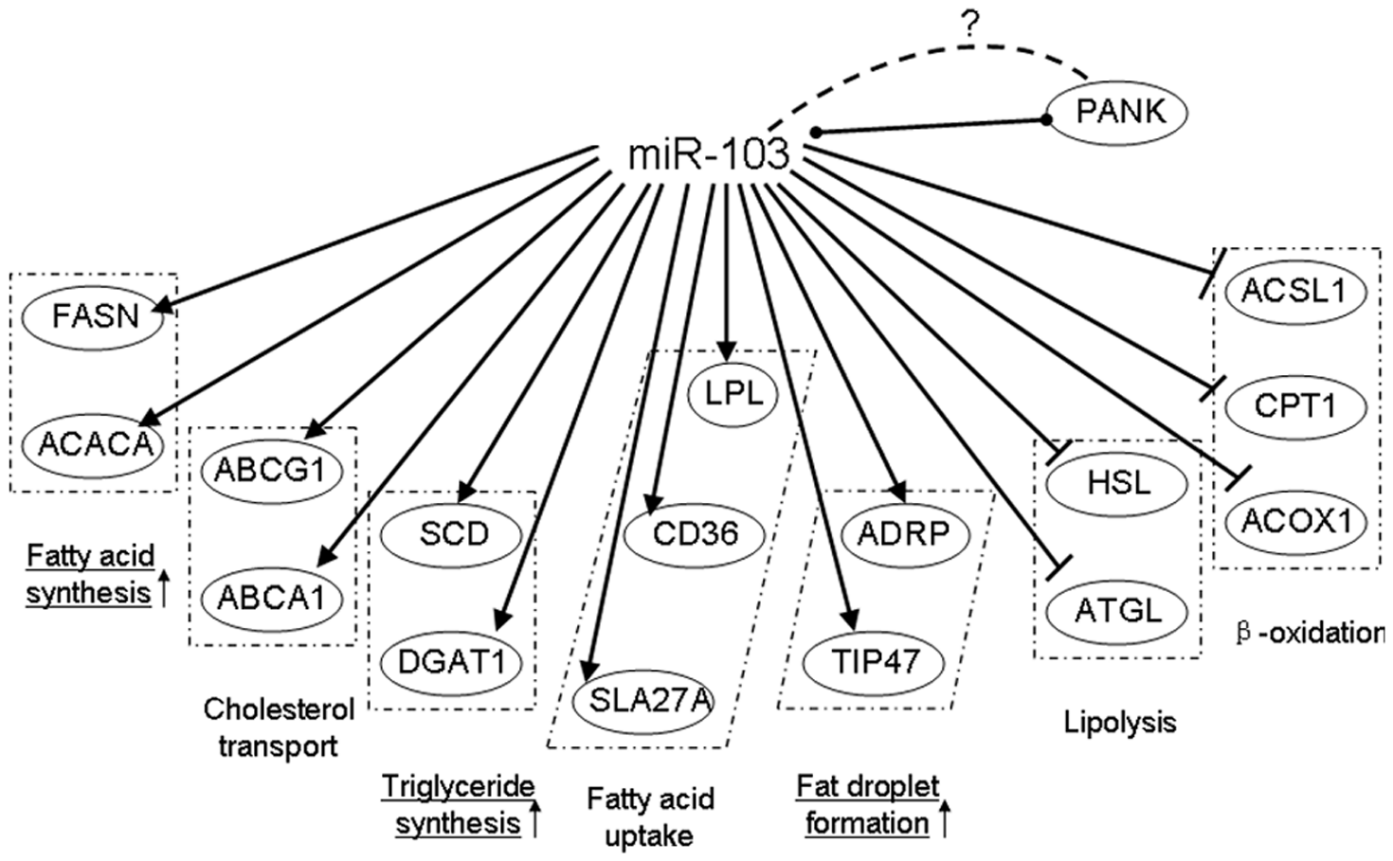
Gene networks modulated by miR-103. Genes associated with milk fat metabolism are shown to be positively (arrowheads) or negatively (end lines) regulated by miR-103 over-expressed. Genes in one box indicated that they were involved in one process of milk fat synthesis. Over-expression of miR-103 can increase fat droplet, triglyceride, and fatty acid contents, which had been identified in the manuscript. MiR-103 was co-regulated with PANK and miR-103 also can regulate PANK expression though an unknown pathway.

For decades, researchers have been dedicated to the study of improving the composition of goat milk to meet the increasingly elaborate nutritional requirements of humans. Currently, the main way to increase the nutritional value of goat milk is by altering their diets [69], [70], [71], for example, either changing their food composition (forage vs concentrate) [72], [73], or adding dietary lipid supplements (i.e., fatty acid), to increase certain component production (i.e., unsaturated fatty acids, conjugated fatty acid in milk) [74], [75]. However, there are numerous drawbacks to this method, such as [76], [77], [78] (1) a limited capacity to produce the desired component of milk through feeding; (2) digestibility of dietary lipid supplements in ruminants does not depend on fatty acid intake (i.e., α-linolenic acid); (3) animal factors (i.e., breed, individuality) affect the fatty acid composition of milk fat. Therefore, to genetically control milk yield and fatty acids composition, we must consider the genetic research [79] or molecular breeding. The first step in molecular breeding for the milk industry is to identify the key genes responsible for milk synthesis. Genome wide association studies [80], [81] and quantitative trait loci (QTL) identification [82], [83] in cows has given us important information about genes related to milk yield and composition, however, the mechanisms in which these key genes control the metabolism and composition of goat milk require further studies. In this study, we determined that miRNAs play crucial role in regulating milk fat synthesis. MiR-103, one of the 30 most abundantly expressed miRNAs in the lactating mammary gland, controls gene expression (Figure 7), goat milk fat accumulation, as well as the ratio of unsaturated/saturated fatty acids. To the best of our knowledge, this is the first evidence that a miRNA controls milk fat synthesis. Our results suggest that miR-103 may be an important regulator for fat composition and nutrient level of goat milk.

Previous studies have shown that normal adipose cells with high triglyceride levels may increase the probability of lipotoxicity [84]. Elevated *PPARγ* expression can prevent lipotoxicity [85]. In our work, not only *PPARγ* expression (Figure 5A), but also the expression of its downstream *ADRP* (Table 1) which is responsible for fat droplet formation [63], were both increased, suggesting that triglyceride in epithelial cells may be used for milk fat droplet formation. Therefore, it eliminated the possibility that the increased triglyceride accumulation in GMEC caused a pathological status.

### MiR-103 may suppress β-oxidation to increase triglyceride content

Triglycerides are not only an important component of milk fat globules, but also a main form of energy storage [66], [67], [68]. Fatty acids from lipolysis are transported to mitochondria where fatty acids undergo β-oxidation for energy production. Fatty acid uptake can also provide material for β-oxidation. All fatty acids will be degraded to acetyl-CoA, some of which will be used for the de novo fatty acid synthesis [66], [68]. The relationship between milk fat synthesis and β-oxidation is shown in many types of cells, such as adipocytes [68] and skeletal muscle cells [86]. In this study, we found *ACSL1* and *ACOX1*, both involved in β-oxidation, to be predicted targets of miR-103 (Table S3). MiR-103 is highly expressed in mid-lactation. Through suppression of *ACSL1* and *ACOX1* expression, miR-103 may decrease the whole β-oxidation level and up-regulate acetyl-CoA amounts for fatty acid and triglyceride synthesis to meet the huge milk demands needed during mid-lactation.

Suppression of β-oxidation resulted in a down-regulation of energy supplies; however Ad-miR-103-infected cells still accumulated more triglycerides. We speculated that the main pattern of cell oxidation for energy supplies might change, such as from β-oxidation to α-oxidation [87]. Furthermore, fat tissue in ruminants is the main energy supply for milk fat synthesis [88]. We found that lipolysis in GMEC is not the main way to provide fatty acids for β-oxidation, for the mRNA expression of lipolysis-related genes (e.g., *HSL* and *ATGL*) are relatively low in normal and Ad-miR-103-infected cells (data not show). In addition, the utilization of fatty acids was higher (the expression of *LPL* and *SLC27A6* was increased, Table 1), indicating that the main origin of fatty acids used for β-oxidation may be from extracellular sources.

MiR-103 may decrease β-oxidation through regulating Leptin (LEP) and AMP-activated protein kinase subunit α (AMPKα) pathways. LEP stimulates lipolysis process [89]. The AMPKα signal pathway directly activates β-oxidation [41], [90]. QRT-PCR analysis (Figure S2) indicated that miR-103 over-expression significantly increased *LEP* mRNA expression relative to controls, while it decreased the expression of *AMPKα*, which is consistent with an increase in fatty acid content and a decrease in β-oxidation levels.

### MiR-103 and its host gene PANK3

Some sense oriented intronic miRNAs are co-regulated with their host genes and positively assist the function of their co-regulated host genes [51], [52]. MiR-103-1 is a sense oriented intronic miRNAs of *PANK3* which plays an important role in generating Acetyl CoA for de novo fatty acid synthesis [49], [50]. In this study, we identified a positive correlation between miR-103-1 and *PANK3* (*P*<0.001, Figure 3A). This correlation between miR-103-1 and *PANK3* expression suggests that the function of miR-103-1 may be related to PANK3's; thereby miR-103 might modulate milk fat synthesis.

De novo fatty acid synthesis and fatty acid uptake are two redundant pathways for supplying fatty acid for milk fat droplet and triglyceride synthesis [56], [57]. Among these two pathways, PANK3 promotes de novo fatty acids synthesis by regulating CoA synthesis [49], [50]; LPL, CD36, and SLC27A6 are involved in fatty acid uptake [56], [57], [58], [59]. In this study, it seems that the fatty acid supplied by PANK is not the major pathway for supplying fatty acid because, when the total amount of fatty acids and triglyceride was increased (Figure 4C, D), *PANK3* expression was down-regulated (Figure 3C), whereas, *LPL*, *CD36* and *SLC27A6* expression involved in fatty acid uptake were up-regulated (Table 1). Therefore, we speculated that fatty acids, through uptake, may be the main origin for the raw material of triglyceride synthesis in the endoplasmic reticulum.

In contrast, to the increased milk fat resulting from miR-103 over-expression, suppression of miR-103 expression had no effect on fat droplet accumulation (data not show). One possible explanation for this is that other miRNAs in the miR-103 family (e.g., miR-107), or synergic miRNAs which target genes in conjunction with miR-103, could regulate fatty acid synthesis, compensating for miR-103 knockdown.

Furthermore, in this study, we identified the relationship between miR-103-1 and *PANK3*. However, we were not able to analyze the relationship between miR-103-2, miR-107 and their host gene *PANK2*, *PANK1*, as the sequences of *PANK1* and *PANK2* in goat are unknown and cDNA cloning did not succeed.

To validate miR-103 target genes, we measured the expression of miR-103 predicted targets (Table S3). The levels of *PDK4*, glutamate dehydrogenase 1(GUD1, data not show), *ACSL1* and *ACOX1* were decreased as miR-103 over-expressed, and their expression were increased as miR-103 expression suppressed (data not show). However, miR-103 expression had little effect on the expression of *JAK1* and other genes. Even so, we have used MFOLD software to analyze the ΔG of the 80 bp flanking sequence of a miR-103 binding site on these predicted targets, and have constructed miR-103 sensors using pGL3-control luciferase reporter vector inserted into the *XbaI* locus with 100-200 bp miRNA binding site of targets. However, we have not yet obtained direct proof that one of these genes is a direct target of miR-103.

Collectively, our data not only provides new insights regarding miRNAs participation in the gene network controlling milk fat synthesis, but also gives us important clues that miR-103 may be used as an index for a molecular breeding program in goats. Furthermore, transgenic goats have been reported to secrete milk containing lactoferrin, α-fetoprotein, and lysozymes [91], [92], [93]. These successes in goats provide important clues that over-expression of certain component are feasible in the mammary gland. Therefore, miR-103 could be a candidate gene used for increasing milk yield or unsaturated fatty acid production in the goat milk industry, which is one of the main objectives of dairy goat breeding.

### Conclusion

In summary, we have identified miR-103 as a new class of regulators of milk fat synthesis. We presented novel data concerning miR-103 expression in lactating goats using a Solexa sequencing approach. For the first time we show that goat mammary gland-enriched miR-103 is linked to milk triglyceride accumulation and unsaturated fatty acids content, indicating that miRNAs may potentially play a role in milk production and the synthesis of beneficial milk components in dairy animal.

## Materials and Methods

### Ethics Statement

The animal care and use were approved by the Institutional Animal Care and Use Committee in the College of Animal Science and Technology, Northwest A&F University, Yangling, China.

### Animals, sampling, and RNA extraction

Dairy goats used in the study were from the elite herd of Xinong Saanen Dairy Goats in the experimental farm of Northwest A&F University of China. 30 healthy, three-year-old goats with similar body weight were selected for this study. All goats given birth the kids in one month were in the second lactation. Mammary gland tissues were surgically collected from the goats at mid-lactation (120 days after parturition) and immediately frozen in liquid nitrogen. All the 30 samples of mammary gland tissue were subjected to total RNA extraction. 10 of the 30 RNA samples were pooled and used for Solexa sequencing. Additionally, mammary gland tissues from 3 of these 30 samples were collected similarly from goats at dry lactation (60 days before parturition). All tissues were weighted 0.5 g–1.0 g.

Total RNA was extracted from the mammary gland tissue using a mirVana miRNA Isolation Kit (Ambion, USA) according to the manufacturer's instructions. The quantity and quality of RNA was measured using a NanoDrop ND-1000 spectrophotometer (Nanodrop, USA). The A260/A280 ratio was >2.1 and the A230/A260 ratio was >1.9 for all samples. Total RNAs were stored at −80°C for further use. We also used a mirVana miRNA Isolation Kit (Ambion, USA) to extract total RNA from GMEC. The A260/A280 ratio was >2.1 and the A230/A260 ratio was >2.0 for all samples.

### Solexa sequencing and bioinformatics analysis for small RNAs

Sequencing RNA samples were pooled from 10 individuals of total mRNA during mid-lactation with equal quantities (10 µg). Subsequently, small RNA library was constructed according to experimental procedure reported previously [32], [33], and was sequenced using Illumina' Genome Analyzer Pipeline software (Illumina, San Diego, USA). The small RNA sequences (18–26 nt) were mapped onto mammal genome. And results were subjected to a series of data filtration step, discarding the sequences matched to rRNA, tRNA, snRNA, snoRNA, repeat sequences, and degraded mRNA. Sequences were retained for miRNA based on several features [94]. Then potential miRNAs were aligned to the statistics of mammalian miRNAs in miRBase 17.0 using the ACGT101-miR program (LC Sciences, Houston, USA). Finally, goat miRNA aligned with other mammalian known miRNAs were obtained. These miRNAs will be referred to conserved miRNAs in this study.

### Primers, cDNA synthesis and qRT-PCR for miRNA and mRNA

For miRNA, first strand cDNA was synthesized using 100 ng total RNA and a TaqMan® MicroRNA Reverse Transcription Kit (Ambion, USA). 100 ng total RNA, 50 U MutiScribeTM Reverse Transcriptase, 3.8 U RNase Inhibitor, 15 mM dNTPs, and 1×Reverse Transcription Buffer were run in a total reaction volume of 15 µl and incubated at 16°C for 30 min, 42°C for 30 min, and 85°C for 5 min. Then, 0.8 µl of the RT reaction was combined with 0.5 µl of and 5 µl of TaqMan® Universal PCR Master Mix in a 10 µl final volume for qPCR. The PCR assay was carried out with a TaqMan MicroR-103 Assay (Ambion, USA) on a Bio-Rad CFX96 real-time PCR detection system (Bio-Rad, USA) with cycling conditions of 95°C for 10 min, followed by 95°C for 15 sec and 60°C for 60 sec for a total of 40 cycles. The miRNA was normalized to U2 or U6 snRNA (TaqMan U2 Assay and U6 assay, Ambion) level. Fold change was determined using the 2^−ΔΔCT^ method.

For mRNA, 1 µg of total RNA was synthesized into cDNA using PrimeScript® RT Reagent Kit (Perfect Real Time, Takara, Japan). RT-reaction volume contained 1 µg total RNA, 50 pmol Oligo dT Primer, 100 pmol Random 6 mers, 200 U PrimeScript® RT Enzyme Mix I, and 1×PrimeScript® Buffer. The mixture of this 20 µl volume was incubated at 37°C for 15 min and at 85°C for 5 min. The first strand cDNA was diluted with DNase/RNase free water. The qPCR assay was performed using SYBR® Premix Ex Taq™ II (Perfect Real Time) (Takara, Japan) on a Bio-Rad CFX96. A total of 20 µl mix composed of 1 µl of RT reaction, 0.4 µM Forward Primer, 0.4 µM Reverse Primer, and 1×SYBR Primix Ex Taq™. This mixture was incubated at 95°C for 30 s, 96°C for 5 s and 60°C for 30 s for a total of 40 cycles. Glyceraldehyde-3-phosphate-dehydrogenase (*GAPDH*) was used as an internal control. MRNA level was normalized to *GAPDH* level. Fold change was determined using 2^-ΔΔCT^ method. Primers features and qPCR performance were listed in Table S4. In addition to the primers from published papers, other primers were designed using goat's genes that were cloned in our laboratory. All primers were synthesized by Invitrogen Corp (USA).

### Cell culture

GMEC was cultured in DMEM/F12 medium (Invitrogen Corp., USA), containing 5 µg/ml insulin, 0.25 µmol/l hydrocortisone, 50 U/ml penicillin/ml streptomycin, 10 ng/ml epidermal growth factor 1 (EGF-1, Gibco), and 10% FBS at 37°C in a humidified atmosphere with 5% CO_2_ [95]. The medium was changed every day. Primary GMEC were isolated under sterile conditions using a modification of the tissue explant method [96]. Briefly, mammary gland epithelial tissue was surgically collected from goat mammary gland at mid-lactation (120 d after parturition) and then rinsed three times with D-Hanks buffer solution containing 1000 U/ml penicillin and 1000 U/ml streptomycin. After removing the connective and adipose tissue that surrounds the mammary epithelium, the mammary gland epithelial tissue was minced into approximately 1 mm^3^ sections and then seeded in tissue culture dishes (35 mm diam.) with 0.5 ml DMEM/F12 medium. The medium was added dropwise between the tissue sections. After 90 min in a humidified atmosphere, the volume of DMEM/F12 medium was brought up to 2 ml and then the dishes were returned to the humidified atmosphere. At confluence, the GMEC were dissociated using Trypsin-EDTA Solution (0.25% Trypsin and 0.05% EDTA). The passage 1 or 2 cells were seeded on DMEM/F12 medium in culture plates (Nunc, Denmark) at a density of 5×10^4^ cells/cm^2^ for adenovirus infection as described below.

### Ad-miR-103 generation and infection

Authentic miR-103 stem-loop and about 300 nucleotides flanking sequences on the 5′ and 3′ side of miR-103 were amplified from normal Xinong Saanen Dairy Goat genomic DNA. The primers for miR-103 stem-loop were designed using Oligo software (version 6.0): forward primer CGCTAGAAGCTTTTGGGTTAA -TACTCCATTGAG, reverse primer: GCCCTAGACCATGGATTTGTCATTTTGTAAAACT. The adenovirus vectors pAd-control(which dose not contain an inserted sequence) and pAd-miR-103 were constructed and packed in HEK 293 cells using a commercial system (AdEasy, Stratagene). GMEC was infected with Ad or Ad-miR-103 at multiplicities of infection MOI (MOI) of 50, 100, 150, 200 or 250. Infection efficiency was determined by observation of green fluorescence under inverted/phase contrast microscopy (Leica CMF-500, Germany). The infection efficiency was highest (70%) at a MOI of 200. MiR-103 expression was higher in Ad-miR-103-infected cells than that in Ad-infected cells, (2.42-fold, *p*<0.05, Figure 3B) at a MOI of 200. For the Ad control, miR-103 in Ad-infected cells (at a MOI of 200) did not change compared to uninfected cells (Figure S3), suggesting that Ad doesn’t alter miR-103 expression by itself at this MOI. Then GMEC at a density of 8×10^4^ cells per well was infected with Ad-miRNAs or Ad at an optimal MOI of 200. The infected cells were cultured for 0, 24, 48, or 72 h and then subjected to RNA extraction and cDNA synthesis as described above. The infected cells for 72 h were also used for the tests described below (Oil Red O staining, triglyceride assay, and fatty acid analysis).

### Transfection GMEC with antisense inhibitor of miR-103

Goat specific miR-103-inhibitor (antisense inhibitor for miR-103) (Anti-miR™ miRNA Inhibitor) and inhibitor negative control antisense oligonucleotide (Anti-miR™ miRNA Inhibitor Negative Control^#^1) were purchased from Invitrogen (USA). The inhibitor control was labeled with FAM fluorescence (Invitrogen, USA). Different doses of inhibitor-control were added to GMEC. And inhibitor-control doesn’t alter miR-103 expression by itself (Figure S3). We found that miR-103 had the lowest expression level with 60 nM inhibitor-control (Figure 3B). Furthermore, the transfection efficiency was nearly 70% (60 nM) by observation of FAM under microscopy. Then, an aliquot (500 µl) of medium in serum-and-antibiotic-free was added to 6-wells incubated with a complex compromising 20 µl transfection reagent, and 60 nM miR-103-inhibitor or 60 nM negative control for 15 min according to procedure manual of Lipofectamine™ RNAiMAX (Invitrogen, USA). Cells were seeded at a density of 2×10^4^ cells per well. RNA was extracted at 48 h after transfection.

### Oil red O staining

The Ad-miRNA-103- and Ad-infected cells cultured in wells (6 well plate) were rinsed three times in phosphate- buffered saline (PBS), fixed in 10% (v/v) paraformaldehyde for 40 min, and then rinsed again with PBS. The fat droplets in the cells were stained with 5% oil red O in isopropanol for 15 min and then examined microscopically. Uninfected cells were also stained for comparison. The culture wells were photographed. Then, we extracted fat droplet and quantitated the fat droplet accumulation according to Sanchez-Hidalgo's method [97]. 100 µl/well of isopropanol were added to each washed and dried stained well, and the stained lipid was allowed to extract for 3 min. The extracted mixture absorbance was read spectrophotometrically at 510 nm using a NanoDrop ND-1000 spectrophotometer (Nanodrop, USA).

### Triglyceride assay

The Ad-miR-103- and Ad-infected cells were harvested in lysis buffer (50 mmol/l Tris-HCL, pH 7.4, 150 mmol/NaCl, 1% Triton X-100) and sonicated to homogenize the cell suspension. Uninfected cells were used as a control. Triglyceride content was measured using a Serum Triglyceride Determination Kit according to the manufacturer's instructions (Sigma–Aldrich).

### Fatty acid extraction and analysis

The way to extract and analyze fatty acids is according to a slightly modified method of Kang and Wang [98]. The Ad-miR-103- and Ad-infected cells were harvested and collected in sealable glass tubes. A 2 ml aliquot of 2.5% (v/v) trichloroacetic acid-methanol solution was added to each tube. The tubes were incubated overnight at 90°C. On the following day, the tubes were cooled to room temperature and then 2 ml saturated KCL solution was added to the tubes followed by 1 ml methyl nonadecanoate (C19:0) (Sigma, no. 74208) as the internal control. The organic compounds in the mixture were extracted twice with 2 ml pure n-hexane. The n-hexane containing fatty acids was transferred to new glass tubes, evaporated to a volume of 500 µl using a nitrogen concentrator HP-5016GD (Ji Cheng Company, Shanghai), and then stored at -20°C. The fatty acid content of the samples was determined using gas chromatography-mass spectrometry (GC-MS) (Agilent 5975, USA) at the Analysis Center of Northwest A&F University. The following conditions were employed: column: HP-5 (60 m×0.25 mm. i.d. ×0.25 µm d.f.); detector temperature: 280°C; split ratio: 1∶10; carrier gas flow: He 0.8 ml/min; injector temperature 250°C; temperature program: starting from 40°C for 2 min, isothermal for 30 min, increasing by 8°C/min to 240°C, then 240°C for 15 min.

### Statistical analysis

Statistical analysis were performed with SPSS software (version 10.0). All data are reported as mean ± standard error (SE). Statistical differences in two groups were determined by Student's *t* test. The differences were considered significant at *p*<0.05, and highly significant as *p*<0.01. RT-qPCR data were analyzed using the ΔΔCT method. Correlation between miR-103-1 and *PANK3* were analyzed using Pearson's correlation coefficient. The figures and Tables shown are representative of at least nine experiments.

## Supporting Information

Figure S1 **Length distribution and frequency of small RNAs.** Small RNAs in goat mammary gland shows an unequally distribution in length. Percentage on each pillar indicates the percentage of miRNA out of the total clean copy number. The majority of small RNA sequences is 21 nt∼23 nt.
(DOC)

Figure S2 **MiR-103 increases** ***LEP*** **expression and decreases** ***AMPKα*** **expression.** Over-expression of miR-103 increases the mRNA expression level of *LEP* (A), whereas decreases the mRNA expression level of *AMPKα* (B). Gene expression in Ad-infected, Ad-miR-103-infected, and uninfected cells was assessed at 0, 24, 48, and 72 h. qRT-PCR measurement of gene expression expressed as fold change compared to their respective level at 0 h. Columns, average of 12 experiments; bars, SEM. *, *p*<0.05. A: *LEP*, leptin protein gene; B: *AMPKα*, AMP-activated protein kinase α.
(DOC)

Figure S3 **MiR-103 expression in GMEC treated with Ad and inhibitor control (treatments are Ad-miR-103 and miR-103 inhibitor).** Ad can make a slight decrease in miR-103 expression at a MOI of ≥300. Inhibitor control has no effect on miR-103 expression at any concentration. The expression levels of miR-103 were determined at 72 h after infecting GMEC with Ad. And the expression levels of miR-103 were determined at 48 h after transfecting GMEC with inhibitor-control. The data (miR-103 levels) were expressed as fold change as compared to normal cells (MOI = 0 and 0 nM), normalized to 1. Columns, average of 3 experiments.
(DOC)

Table S1 **Small RNAs length distribution and frequency in goat mammary gland at mid-lactation.** All small RNAs were mapped to GenBank, Rebase, and miRBase, and then classified as degraded mRNA, tRNA, rRNA, sno/snRNA, other non-coding RNAs and unannotation. The percentage of copy number of RNAs in total copy number (24,479,296) was calculated.
(DOC)

Table S2 **Most abundant miRNAs in mammary gland of lactating goats.**
(DOC)

Table S3 **Predicted targets of miR-103.**
(DOC)

Table S4 **Feature of mRNA primers for qPCR analysis.**
(DOC)
