# Supplementary figures and images for: MiR-103 Controls Milk Fat Accumulation in Goat (Capra hircus) Mammary Gland during Lactation
Source: PLoS One. 2013 Nov 11;8(11):e79258. doi: 10.1371/journal.pone.0079258 (PMC3823599; doi:10.1371/journal.pone.0079258)

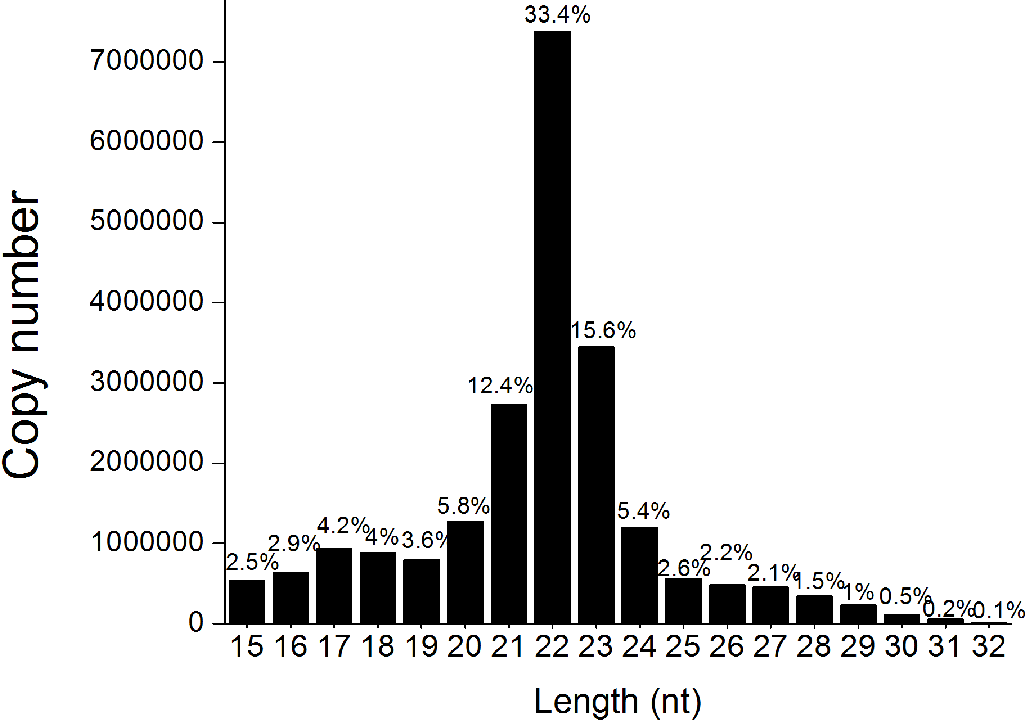

Supplement: Figure S1 — Length distribution and frequency of small RNAs. Small RNAs in goat mammary gland shows an unequally distribution in length. Percentage on each pillar indicates the percentage of miRNA out of the total clean copy number. The majority of small RNA sequences is 21 nt∼23 nt. (DOC) [file pone.0079258.s001.doc]

**
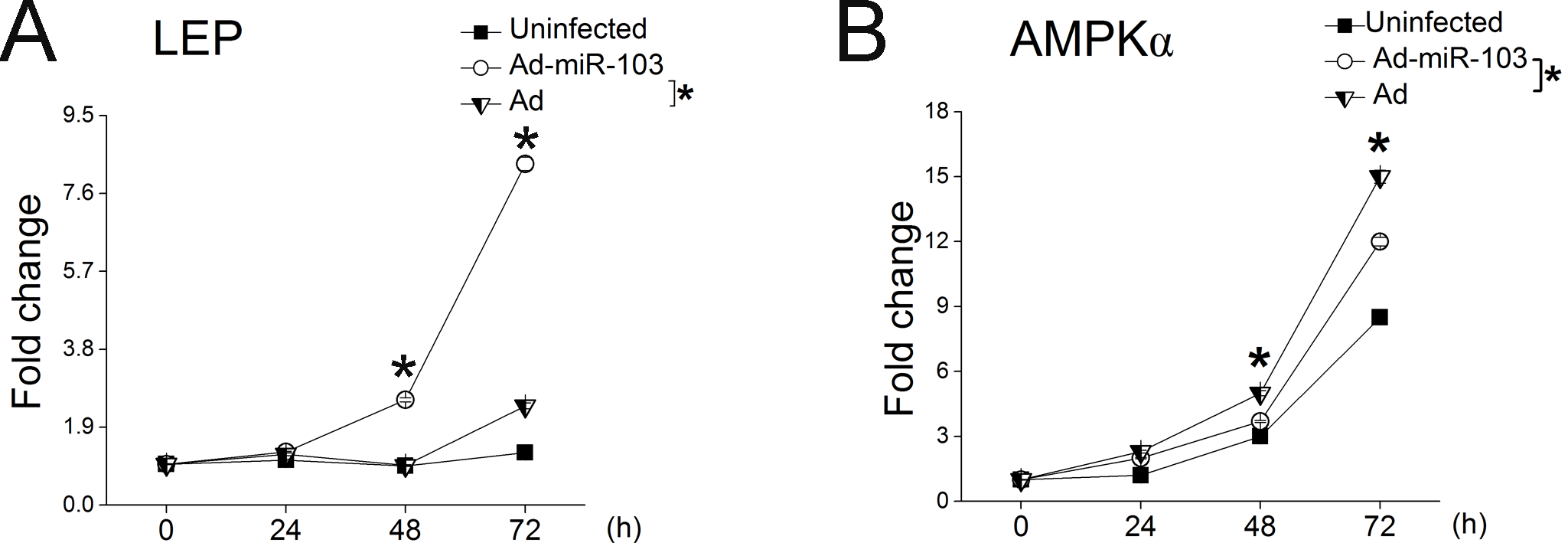
**

Supplement: Figure S2 — MiR-103 increases LEP expression and decreases AMPKα expression. Over-expression of miR-103 increases the mRNA expression level of LEP (A), whereas decreases the mRNA expression level of AMPKα (B). Gene expression in Ad-infected, Ad-miR-103-infected, and uninfected cells was assessed at 0, 24, 48, and 72 h. qRT-PCR measurement of gene expression expressed as fold change compared to their respective level at 0 h. Columns, average of 12 experiments; bars, SEM. *, p<0.05. A: LEP, leptin protein gene; B: AMPKα, AMP-activated protein kinase α. (DOC) [file pone.0079258.s002.doc]

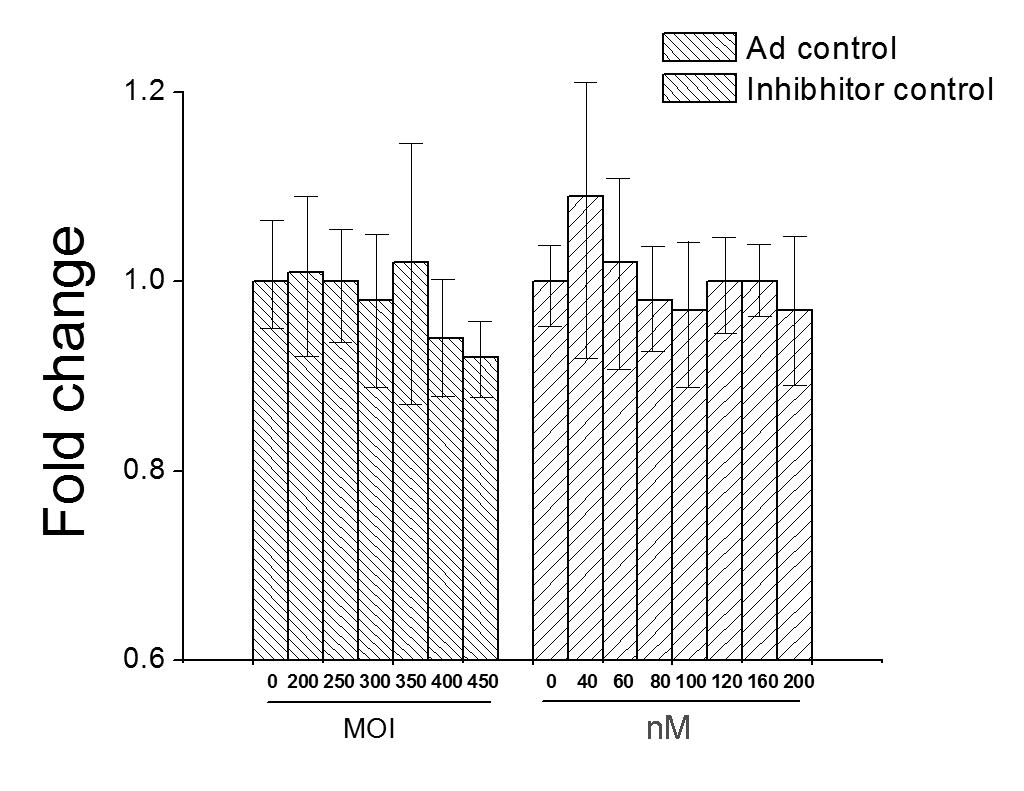

Supplement: Figure S3 — MiR-103 expression in GMEC treated with Ad and inhibitor control (treatments are Ad-miR-103 and miR-103 inhibitor). Ad can make a slight decrease in miR-103 expression at a MOI of ≥300. Inhibitor control has no effect on miR-103 expression at any concentration. The expression levels of miR-103 were determined at 72 h after infecting GMEC with Ad. And the expression levels of miR-103 were determined at 48 h after transfecting GMEC with inhibitor-control. The data (miR-103 levels) were expressed as fold change as compared to normal cells (MOI = 0 and 0 nM), normalized to 1. Columns, average of 3 experiments. (DOC) [file pone.0079258.s003.doc]
