# Supplementary material for: MiR-103 Controls Milk Fat Accumulation in Goat (Capra hircus) Mammary Gland during Lactation
Source: PLoS One. 2013 Nov 11;8(11):e79258. doi: 10.1371/journal.pone.0079258 (PMC3823599; doi:10.1371/journal.pone.0079258)
Supplement: Table S1 — Small RNAs length distribution and frequency in goat mammary gland at mid-lactation. All small RNAs were mapped to GenBank, Rebase, and miRBase, and then classified as degraded mRNA, tRNA, rRNA, sno/snRNA, other non-coding RNAs and unannotation. The percentage of copy number of RNAs in total copy number (24,479,296) was calculated. (DOC) [file pone.0079258.s004.doc]

| **Categories of small RNAs** | **Copy number of Solexa** | **Percentage** |
| --- | --- | --- |
| MiRNA | 12,367,141 | 47.7 % |
| MRNA | 1,788,957 | 6.9 % |
| rRNA,soRNA, snRNA, etc | 2,126,007 | 8.20 % |
| Mapped to Repbase | 270,433 | 1.2 % |
| Unannotation | 9,333,691 | 36 % |
| Total clean reads | 24,479,296 | 100 % |
