# Supplementary material for: MiR-103 Controls Milk Fat Accumulation in Goat (Capra hircus) Mammary Gland during Lactation
Source: PLoS One. 2013 Nov 11;8(11):e79258. doi: 10.1371/journal.pone.0079258 (PMC3823599; doi:10.1371/journal.pone.0079258)
Supplement: Table S2 — Most abundant miRNAs in mammary gland of lactating goats. (DOC) [file pone.0079258.s005.doc]

| **MiRNA** | **Reads** | **Sequence (5′**-**3′)** | **Length** | **Reference** |
| --- | --- | --- | --- | --- |
| **Lipid metabolism** |  |  |  |  |
| bta-miR-23a | 48467 | ATCACATTGCCAGGGATTTCCA | 22 | [1] |
| bta-miR-27b | 157649 | TTCACAGTGGCTAAGTTCTGC | 21 | [2] |
| bta-miR-200a_R-1 | 112157 | TAACACTGTCTGGTAACGATGT | 22 | [3] |
| bta-miR-200b_R+1 | 195203 | TAATACTGCCTGGTAATGATGA | 22 | [3] |
| bta-miR-200c | 105304 | TAATACTGCCGGGTAATGATGGA | 23 | [3] |
| bta-miR-103 | 272319 | AGCAGCATTGTACAGGGCTATGA | 23 | [4][5][6] |
| **Confer cell phenotype** |  |  |  |  |
| bta-miR-30a-5p | 314708 | TGTAAACATCCTCGACTGGAAGCT | 24 | [7] |
| bta-miR-30d | 106718 | TGTAAACATCCCCGACTGGAAGCT | 24 | [7] |
| bta-miR-29a_L-1R-1 | 68797 | TAGCACCATCTGAAATCGGTT | 21 | [8] |
| **Proliferation and apoptosis** |  |  |  |  |
| bta-miR-21_R-2 | 455788 | TAGCTTATCAGACTGATGTTGA | 22 | [9][10] |
| bta-miR-26a | 89789 | TTCAAGTAATTCAGGATAGGTT | 22 | [11] |
| bta-miR-26b | 584912 | TTCAAGTAATCCAGGATAGGCT | 22 | [11] |
| bta-miR-99a | 70869 | AACCCGTAGATCCGATCTTGT | 22 | [12] |
| bta-miR-205 | 124795 | TCCTTCATTCCACCGGAGTCTG | 22 | [13] |
| bta-miR-22-3p_R+1 | 52547 | AAGCTGCCAGTTGAAGAACTGT | 22 | [14] |
| bta-miR-25 | 72594 | CATTGCACTTGTCTCGGTCTGA | 22 | [15] |
| bta-miR-375_L-1 | 72221 | TTTGTTCGTTCGGCTCGCGTGA | 22 | [16] |
| bta-miR-191 | 88842 | CAACGGAATCCCAAAAGCAGCTG | 23 | [17] |
| bta-miR-125b | 45365 | TCCCTGAGACCCTAACTTGTGA | 22 | [18] |
| bta-miR-423-3p_L-1 | 55393 | AGCTCGGTCTGAGGCCCCTCAGT | 23 | [19] |
| **Immune response and development** |  |  |  |  |
| bta-miR-181a_R-1 | 78137 | AACATTCAACGCTGTCGGTGAGT | 23 | [20][21] |
| bta-let-7a | 321661 | TGAGGTAGTAGGTTGTATAGTT | 22 | [22] |
| bta-let-7b | 357712 | TGAGGTAGTAGGTTGTGTGGTT | 22 | [20][21]] |
| bta-let-7f | 281644 | TGAGGTAGTAGATTGTATAGTT | 22 | [21] |
| bta-let-7g | 190817 | TGAGGTAGTAGTTTGTACAGTT | 22 | [20][21] |
| bta-let-7i | 215538 | TGAGGTAGTAGTTTGTGCTGTT | 22 | [20][21] |
| **Controls insulin content** |  |  |  |  |
| bta-miR-148a | 481189 | TCAGTGCACTACAGAACTTTGT | 22 | [23] |
| **Highly expressed in adipose tissue** |  |  |  |  |
| bta-miR-143_R-1 | 348389 | TGAGATGAAGCACTGTAGCTC | 21 | [24] |
| bta-miR-145 | 93153 | GTCCAGTTTTCCCAGGAATCCCT | 23 | [25] |
| **Function unkown** |  |  |  |  |
| bta-miR-151* | 120455 | TCGAGGAGCTCACAGTCTAGT | 21 |  |

# References

# 1. [Frost](http://www.springerlink.com/content/?Author=Robert+J.+A.+Frost) RJA, [Rooij](http://www.springerlink.com/content/?Author=Eva+van+Rooij) EV. (2010) [miRNAs as Therapeutic Targets in Ischemic Heart Disease](http://www.springerlink.com/content/l5h62221170835m6/). [J Cardiovasc Translational Res](http://www.springerlink.com/content/1937-5387/) 3: 280–28.

# 2. Lin Q, Gao ZG, Alarcon RM. (2009) A role of miR-27 in the regulation of adipogenesis. FEBS J 276: 2348–2358.

# 3. Teleman AA. (2010) miR-200 De-FOGs insulin signaling. Cell Metab 11:8–9.

# 4. Trajkovski M, Hausser J, Soutschek J, Bhat B, Akin A, et al. (2011) MicroRNAs 103 and 107 regulate insulin sensitivity. Nature 474: 649–654.

# 5. Wilfred BR, Wang WX, Nelson PT. (2007) Energizing miRNA research: A review of the role of miRNAs in lipid metabolism, with a prediction that miR-103/107 regulates human metabolic pathways. Mol Genet Metab [91: 209–217](http://www.ncbi.nlm.nih.gov/entrez/eutils/elink.fcgi?dbfrom=pubmed&retmode=ref&cmd=prlinks&id=17521938).

# 6. Xie HM, Lim B, Lodish HF. (2009) MicroRNAs Induced During Adipogenesis that Accelerate Fat Cell Development Are Down regulated in Obesity. Diabetes 58:1050–1057.

# 7. [Joglekar MV](http://www.ncbi.nlm.nih.gov/pubmed?term="Joglekar MV"%5BAuthor%5D), [Patil D](http://www.ncbi.nlm.nih.gov/pubmed?term="Patil D"%5BAuthor%5D), [Joglekar VM](http://www.ncbi.nlm.nih.gov/pubmed?term="Joglekar VM"%5BAuthor%5D), [Rao GV](http://www.ncbi.nlm.nih.gov/pubmed?term="Rao GV"%5BAuthor%5D), [Reddy DN](http://www.ncbi.nlm.nih.gov/pubmed?term="Reddy DN"%5BAuthor%5D), et al. (2009) The miR-30 family microRNAs confer epithelial phenotype to human pancreatic cells. PubMed 1:137–47.

# 8. Gebeshuber CA, Zatloukal K, Martinez J. (2009) miR-29a suppresses tristetraprolin, which is a regulator of epithelial polarity and metastasis. EMBO reports 10: 400–405.

# 9. [Frankel](http://www.jbc.org/search?author1=Lisa+B.+Frankel&sortspec=date&submit=Submit) LB, [Christoffersen](http://www.jbc.org/search?author1=Nanna+R.+Christoffersen&sortspec=date&submit=Submit) NR, [Jacobsen](http://www.jbc.org/search?author1=Anders+Jacobsen&sortspec=date&submit=Submit) A, [Lindow](http://www.jbc.org/search?author1=Morten+Lindow&sortspec=date&submit=Submit) M, [Krogh](http://www.jbc.org/search?author1=Anders+Krogh&sortspec=date&submit=Submit) A, et al. (2008) Programmed Cell Death 4 (PDCD4) Is an Important Functional Target of the MicroRNA miR-21 in Breast Cancer Cells. J Bio.Chem 283: 1026–1033.

# 10. Kulshreshtha R , Davuluri RV , Calin GA , Ivan M. (2008) A microRNA component of the hypoxic response. Cell Death Differ 15：667–671.

11. Huse JT, Brennan C, Hambardzumyan D, Wee B, Pena J, et al. (2009) The PTEN-regulating microRNA miR-26a is amplified in high-grade glioma and facilitates gliomagenesis in vivo. Genes & Dev 23: 1327-1337.

# 12. [Sun](http://cancerres.aacrjournals.org/search?author1=Dandan+Sun&sortspec=date&submit=Submit) DD, [Lee](http://cancerres.aacrjournals.org/search?author1=Yong+Sun+Lee&sortspec=date&submit=Submit) YS, [Malhotra](http://cancerres.aacrjournals.org/search?author1=Ankit+Malhotra&sortspec=date&submit=Submit) A, [Kim](http://cancerres.aacrjournals.org/search?author1=Hak+Kyun+Kim&sortspec=date&submit=Submit) HK, [Matecic](http://cancerres.aacrjournals.org/search?author1=Mirela+Matecic&sortspec=date&submit=Submit) M, et al. (2011) miR-99 Family of MicroRNAs Suppresses the Expression of Prostate-Specific Antigen and Prostate Cancer Cell Proliferation. Cancer Res 71: 1313.

# 13. Wu HL, Zhu SM, Mo YY. (2009) Suppression of cell growth and invasion by miR-205 in breast cancer. Cell Res 19:439–448.

# 14. Ting Y, Medina DJ, Strair RK, Schaar DG. (2010) Differentiation-associated miR-22 represses Max expression and inhibits cell cycle progression. [BBRC](http://www.sciencedirect.com/science/journal/0006291X) 394: 606–611.

# [15. Petrocca](http://cancerres.aacrjournals.org/search?author1=Fabio+Petrocca&sortspec=date&submit=Submit) F, [Vecchione](http://cancerres.aacrjournals.org/search?author1=Andrea+Vecchione&sortspec=date&submit=Submit) A, Croce CM. (2008) Emerging Role of miR-106b-25/miR-17- 92 Clusters in the Control of Transforming Growth Factor β Signaling. Cancer Res 68: 8191.

# 16. Ding L, Xu YJ, Zhang W, Deng Y, Si MS, et al. (2010) MiR-375 frequently downregulated in gastric cancer inhibits cell proliferation by targeting JAK2. Cell Res 20:784–793.

# 17. [Elyakim](http://cancerres.aacrjournals.org/search?author1=Eran+Elyakim&sortspec=date&submit=Submit) E, [Sitbon](http://cancerres.aacrjournals.org/search?author1=Einat+Sitbon&sortspec=date&submit=Submit) E, [Faerman](http://cancerres.aacrjournals.org/search?author1=Alexander+Faerman&sortspec=date&submit=Submit) A, [Tabak](http://cancerres.aacrjournals.org/search?author1=Sarit+Tabak&sortspec=date&submit=Submit) S, [Montia](http://cancerres.aacrjournals.org/search?author1=Eve+Montia&sortspec=date&submit=Submit) E, et al. (2010) Hsa-miR-191 Is a Candidate Oncogene Target for Hepatocellular Carcinoma Therapy. Cancer Res 70: 8088–8087.

# 18. Xia HF, He TZ, Liu CM, Cui Y, Song PP, et al. (2009) MiR-125b expression affects the proliferation and apoptosis of human glioma cells by targeting BMF. Cell Physiol Biochem 23:347–358

# 19. Boren[a](http://www.sciencedirect.com/science/article/pii/S009082580800200X#aff1%23aff1) T, Xiong[a](http://www.sciencedirect.com/science/article/pii/S009082580800200X#aff1%23aff1) Y, Hakam[d](http://www.sciencedirect.com/science/article/pii/S009082580800200X#aff4%23aff4) A, Wenham[a](http://www.sciencedirect.com/science/article/pii/S009082580800200X#aff1%23aff1) R, Apte[a](http://www.sciencedirect.com/science/article/pii/S009082580800200X#aff1%23aff1) S, et al. (2008) MicroRNAs and their target messenger RNAs associated with endometrial carcinogenesis. [Gynecologic Oncology](http://www.sciencedirect.com/science/journal/00908258) 110: 206–215.

# 20. Sonkoly E, Stahle M, Pivarcsi A. (2008) MicroRNAs and immunity: novel players in the regulation of normal immune function and inflammation. Semin Cancer Biol 18:131–140.

# 21. Lodish HF, Zhou BY, Liu GW, Chen CZ. (2008) Micromanagement of the immune system by microRNAs. Nat Rev Immun 8: 120–130.

# 22. Yu FY, Yao HR, Zhu PC, Zhang QQ, Pan QH, et al. (2007) let-7 Regulates Self Renewal and Tumorigenicity of Breast Cancer Cells. Cell 131: 1109–1123.

# 23. Melkman-Zehavi T, Oren R, Kredo-Russo S, Shapira T, Mandelbaum AD, et al. (2011) miRNAs control insulin content in pancreatic β-cells via downregulation of transcriptional repressors. EMBO J 30: 835–845.

# 24. Gu ZL, Eleswarapu S, Jiang HL. (2007) Identification and characterization of microRNAs from the bovine adipose tissue and mammary gland. FEBS letter 581: 981–988.

25. Tang XQ, Tang GL, Özcan S. (2008) Role of microRNAs in diabetes. [BBA](http://www.sciencedirect.com/science/journal/18749399) 1779: 697–701.
