# Supplementary material for: MiR-103 Controls Milk Fat Accumulation in Goat (Capra hircus) Mammary Gland during Lactation
Source: PLoS One. 2013 Nov 11;8(11):e79258. doi: 10.1371/journal.pone.0079258 (PMC3823599; doi:10.1371/journal.pone.0079258)
Supplement: Table S3 — Predicted targets of miR-103. (DOC) [file pone.0079258.s006.doc]

| **Gene symbol ^1^** | **Full name** | **Database ^2^** | **Function** |
| --- | --- | --- | --- |
| ACOX1 | Acyl-CoA oxidase 1 | Microcosm, PicTar | first step for β-oxidation [1] |
| MAP3K2 | Threonine kinases | PicTar | threonine phosphorylation pathway [2] |
| ACSL1 | Long-chain acyl-CoA synthetase 1 | PicTar | transport fatty acid [3] |
| PDK4 | Pyruvate dehydrogenase kinase 4 | Microcosm, TargetScan | regulating [pyroracemic](app:ds:pyroracemic) [acid](app:ds:acid) metabolism [4] |
| GLUD1 | Glutamate dehydrogenase 1 | TargetScan | glucose transport [5] |
| JAK1 | Janus kinases 1 | TargetScan | phosphorylation of STAT [6] |

^1^ These genes are human mRNA. All these genes are predicted miR-103 target genes

**^2^** TargetScan (Release 6.2, 2012), PicTar (Release 3.26, 2007), and MicroCosm (Release 8.1, 2012)

Databases for target prediction don’t include goat sequences. We predicted miR-103 target by using human mRNA database.

# Reference

# 1. Jeppesen J, Kiens B. (2012) Regulation and limitations to fatty acid oxidation during exercise. J Physiology 590: 1059–1068.

# 2. Yan GR, Chen NP, Huang YD, Ding W, He GW, et al. (2010) Signaling Networks in Gastric Cancer Cells Revealed by Phosphoproteomics. J Proteomics Bioinform 3: 113–120.

# 3. [Li](http://www.jbc.org/search?author1=Lei+O.+Li&sortspec=date&submit=Submit) LO, [Ellis](http://www.jbc.org/search?author1=Jessica+M.+Ellis&sortspec=date&submit=Submit) JM, [Paich](http://www.jbc.org/search?author1=Heather+A.+Paich&sortspec=date&submit=Submit) HA, [Wang](http://www.jbc.org/search?author1=Shuli+Wang&sortspec=date&submit=Submit) SL, [Gong](http://www.jbc.org/search?author1=Nan+Gong&sortspec=date&submit=Submit) N, et al. (2009) Liver-specific Loss of Long Chain Acyl-CoA Synthetase-1 Decreases Triacylglycerol Synthesis and β-Oxidation and Alters Phospholipid Fatty Acid Composition. J Biol Chem 284: 27816–27826.

# 4. Hwang B, Wu PF, Harris RA. (2012) Additive effects of clofibric acid and pyruvate dehydrogenase kinase isoenzyme 4 (PDK4) deficiency on hepatic steatosis in mice fed a high saturated fat diet. FEBS J 279: 1883–1893.

# 5. [Bell GI,Kayano T, Buse JB. (1990) Molecular biology of mammalian glucose transporters. Diabetes Care 13: 198–208.](http://scholar.google.com/scholar?q=+Molecular+biology+of+mammalian+glucose+transporters+&hl=zh-CN&btnG=搜索)

6. [Wang LH,Kirken RA,Erwin RA, et al. (1999) JAK1,STAT,and MAPK signaling pathways as novel molecular targets for the tyrphostin AG490 regulation of IL-2-mediated T cell response. Immun 162: 3897–904.](http://scholar.google.com/scholar?q=JAK1%2CSTAT%2Cand+MAPK+signaling+pathways+as+novel+molecular+targets+for+the+tyrphostin+AG490+regulation+of+IL-2-mediated+T+cell+response&hl=zh-CN&btnG=搜索)
