# Supplementary material for: MiR-103 Controls Milk Fat Accumulation in Goat (Capra hircus) Mammary Gland during Lactation
Source: PLoS One. 2013 Nov 11;8(11):e79258. doi: 10.1371/journal.pone.0079258 (PMC3823599; doi:10.1371/journal.pone.0079258)
Supplement: Table S4 — Feature of mRNA primers for qPCR analysis. (DOC) [file pone.0079258.s007.doc]

GenBank accession number, primer sequence, and amplicon size.

| **Gene** | **Accession #** | **Primers (5′**∼**3′)^1^** | **Bp^2^** | **Source** |
| --- | --- | --- | --- | --- |
| ***FASN*** | **DQ915966** | F: GTCGTTGTCTACAGCACAGCCT | 155 | This manuscript |
|  |  | R: ATGGCGAGGTTCCACTCAAAC |  |  |
| ***ACACA*** | **DQ370054.1** | F: CATCTTGTCCGAAACGTCGAT | 91 | [1] |
|  |  | R: CCCTTCGAACATACACCTCCA |  |  |
| ***SCD*** | **GU947654** | F: CCATCGCCTGTGGAGTCAC | 243 | This manuscript |
|  |  | R: GTCGGATAAATCTAGCGTAGCA |  |  |
| *GPAM* | AY515690 | F: GCAGGTTTATCCA GTATGGCATT | 63 | [1] |
|  |  | R: GGACTGATATCTTCCTGATCATCTTG |  |  |
| *AGPAT6* | NM_00108366.1 | F: CAAAAGACCCCACGTCACT | 201 | This manuscript |
|  |  | R: CTCAGCAGGTTCCAGGACTC |  |  |
| *LPIN1* | NM_00120615.1 | F: TCCCTGCTCGGACGTAATTG | 111 | This manuscript |
|  |  | R: TGGCCACCAGA ATAAAGCATG |  |  |
| ***DGAT1*** | **HM566448** | F: CCACTGGGACCTGAGGTGTC | 123 | This manuscript |
|  |  | R: GCATCACCACACACCAATTCA |  |  |
| ***ADFP*** | **HQ846827** | F: CCCCAGAAGCCGAGTTACTATGTT | 316 | This manuscript |
|  |  | R: CACGCAGCCAGGACAGATAGAG |  |  |
| ***TIP47*** | **HQ846826** | F: GGTGGAGGGTCAGGAGAAA | 114 | This manuscript |
|  |  | R: TCACGGAACATGGCGAGT |  |  |
| ***gBTN1A1*** | **EF102891** | F: TCACGAGGGAGAGGAGTTTC | 165 | This manuscript |
|  |  | R: GGAAGAAGGATGCTGGTATG |  |  |
| ***LPL*** | **DQ997818** | F: GCCAAAAGAAGCAGCAAGATG | 196 | This manuscript |
|  |  | R: CCGAGCGAAGTAGAAAGGAGTATG |  |  |
| ***CD36*** | **JF690773.1** | F: GTACAGATGCAGCCTCATTTCC | 105 | [1] |
|  |  | R: TGGACCTGCAAATATCAGAGGA |  |  |
| *SLC27A6* | NM_00110116.1 | F: CCAAGACTCCCAGAAGGT | 338 | This manuscript |
|  |  | R: GGCTGTTGTTCCAGAAGTAA |  |  |
| *ABCA1* | NM_00102469.1 | F: CGGCGGCTTCTCTTGTATA GC | 101 | [1] |
|  |  | R: TTCAAGCGTGAGCTGAAACG |  |  |
| ***ABCG1*** | **GQ241418.1 GI** | F: CGTCCATAGGTTTCCACTGTGT | 105 | This manuscript |
|  |  | R: GCACAGCAGAAGAATCTCCATA |  |  |
| *GAPDH* | AJ431207.1 | F; GCAAGTTCCACGGCACAG | 229 | This manuscript |
|  |  | R: GGTTCACGCCCATCACAA |  |  |
| ***A-FABP*** | **AY466498** | F: CCTTCAAATTGGGCCAGGA | 148 | This manuscript |
|  |  | R: CAGCACCAGCTTATCATCCAC |  |  |

^1^ Primer sequence (5′∼ 3′) (F – forward; R – reverse);

^2^ Amplicon size in base pair (bp);

Bold symbols mean that this gene was cloned in our lab from goat.

**(Cont.)**

GenBank accession number, primer sequence, and amplicon size.

| **Gene** | **Accession #** | **Primers (5′**∼**3′)^1^** | **bp^2^** | **Source** |
| --- | --- | --- | --- | --- |
| *ACLY* | NM_00103745.1 | F: ACACACTCAGGAGGAGGAGTT | 304 | This manuscript |
|  |  | R: CGCTCAAGGTAGGTGAAGTACAG |  |  |
| *PANK3* | NM_00107546.1 | F: TTATGAAAGATTTGGTCTGCC | 257 | This manuscript |
|  |  | R: ATAACGCTTTCAGTTGACCTT |  |  |
| *ACSS1* | NM_174746.2 | F: CCGATCAGGTCCTGGTAGTGA | 190 | This manuscript |
|  |  | R: CTCGGCCCATGACAATCTTC |  |  |
| *PDK4* | NM_00110188.1 | F: GCCTAACACAAGTGAATGTAAAA | 108 | This manuscript |
|  |  | R: AACTGAAGAGACGGTCAATGA |  |  |
| ***GPR41*** | **HM013824** | F: CGCATTCTTCACCACCATCT | 181 | This manuscript |
|  |  | R: GCAGGTCCCGTTGATACC |  |  |
| ***PPARγ*** | **HQ589347** | F: TCCGTGATGGAAGACCACTC | 308 | This manuscript |
|  |  | R: CCCTTGCATCCTTCACAAGC |  |  |
| ***SREBP-1c*** | **JN790254.1** | F: CCAGCTGACAGCTCCATTGA | 175 | This manuscript |
|  |  | R: TGCGCGCCACAAGGA |  |  |
| ***LXRα*** | **GU332719** | F: CATCAACCCCATCTTCGAGTT | 161 | This manuscript |
|  |  | R: CAGGGCCTCCACATATGTGT |  |  |
| ***HSL*** | **EU273879** | F: GGGAGCACTACAAACGCAACG | 226 | This manuscript |
|  |  | R: TGAATGATCCGCTCAAACTCG |  |  |
| ***ATGL*** | **GQ918145** | F: GGAGCTTATCCAGGCCAATG | 118 | This manuscript |
|  |  | R: TGCGGGCAGATGTCACTCT |  |  |
| *ACSL1* | NM_00107608.1 | F: TGACTGTTGCTGGAGACTGG | 220 | This manuscript |
|  |  | R: CAGCCGTCTTTATCCAGAGC |  |  |
| *CPT1* | FJ415874.1 | F: AAGGACCTCTACGCCAACACG | 225 | [2] |
|  |  | R: TTTGCGGTGGACGATGGAG |  |  |
| *PPARα* | NM_00103403.1 | F: TACTCTCGGCAGACTTCCTAC | 201 | This manuscript |
|  |  | R: CCTCCTCACATCTGTCATACAC |  |  |
| *ACOX1* | NM_00103528.2 | F: CGAGTTCATTCTCAACAGTCCT | 211 | This manuscript |
|  |  | R: GCATCTTCAAGTAGCCATTATCC |  |  |
| *AMPKα* | NM_00102455.1 | F: GGAGCTTATCCAGGCCAATG | 314 | [2] |
|  |  | R: TGCGGGCAGATGTCACTC |  |  |
| *PANK3* | NM_001075463.1 | F: TTATGAAAGATTTGGTCTGCC | 267 | This manuscript |
|  |  | R: ATAACGCTTTCAGTTGACCTT |  |  |
| *LEP* | NM_173928.2 | F: AGGGTCACTGGCTTGGACTTCATC | 271 | This manuscript |
|  |  | R: CGTGGATCTGTTGGTAGATTGCC |  |  |

^1^ Primer sequence (5′∼3′) (F – forward; R – reverse);

^2^ Amplicon size in base pair (bp);

Bold symbols mean that this gene was cloned from goat.

**Reference**

# 1. Bionaz M, Loor JJ. (2008) Gene networks driving bovine milk fat synthesis during the lactation cycle. BMC Genomics 9:366.

# 2. Zhang N, Li QZ, Gao XJ, Yan HB. (2011) Potential role of adenosine monophosphate-activated protein kinase in regulation of energy metabolism in dairy goat mammary epithelial cells. J Dairy Sci 94: 218–222.
